# Stirring the hydrogen and butanol production from Enset fiber via simultaneous saccharification and fermentation (SSF) process

**DOI:** 10.1186/s40643-024-00809-w

**Published:** 2024-10-10

**Authors:** Nebyat Seid, Lea Wießner, Habibu Aliyu, Anke Neumann

**Affiliations:** 1 Electrobiotechnology, Institute of Process Engineering in Life Science 2, Karlsruhe Institute of Technology (KIT), 76131 Karlsruhe, Germany; 2 School of Chemical and Bio Engineering, Addis Ababa Institute of Technology, Addis Ababa University, P.O.B: 1176, Addis Ababa, Ethiopia; 3 Institute for Biological Interfaces 5, Karlsruhe Institute of Technology (KIT), 76344 Karlsruhe, Germany

**Keywords:** Hydrogen, Butanol, Enset fiber, *C. saccharoperbutylacetonicum*, SSF, PSSF

## Abstract

Enset fiber is a promising feedstock for biofuel production with the potential to reduce carbon emissions and improve the sustainability of the energy system. This study aimed to maximize hydrogen and butanol production from Enset fiber through simultaneous saccharification and fermentation (SSF) process in bottles as well as in bioreactor. The SSF process in bottles resulted in a higher butanol concentration of 11.36 g/L with a yield of 0.23 g/g and a productivity of 0.16 g/(L h) at the optimal process parameters of 5% (w/v) substrate loading, 16 FPU/g cellulase loading, and 100 rpm agitation speed from pretreated Enset fiber. Moreover, a comparable result to the bottle experiment was observed in the bioreactor with pH-uncontrolled SSF process, although with a decreased in butanol productivity to 0.095 g/(L h). However, using the pre-hydrolysis simultaneous saccharification and fermentation (PSSF) process in the bioreactor with a 7% (w/v) substrate loading led to the highest butanol concentration of 12.84 g/L with a productivity of 0.104 g/(L h). Furthermore, optimizing the SSF process parameters to favor hydrogen resulted in an increased hydrogen yield of 198.27 mL/g-Enset fiber at atmospheric pressure, an initial pH of 8.0, and 37 °C. In general, stirring the SSF process to shift the product ratio to either hydrogen or butanol was possible by adjusting temperature and pressure. At 37 °C and atmospheric pressure, the process resulted in an e-mol yield of 12% for hydrogen and 38% for butanol. Alternatively, at 30 °C and 0.55 bar overpressure, the process achieved a yield of 6% e-mol of hydrogen and 48% e-mol of butanol. This is the first study to produce hydrogen and butanol from Enset fiber using the SSF process and contributes to the development of a circular bioeconomy.

**Graphical Abstract:**

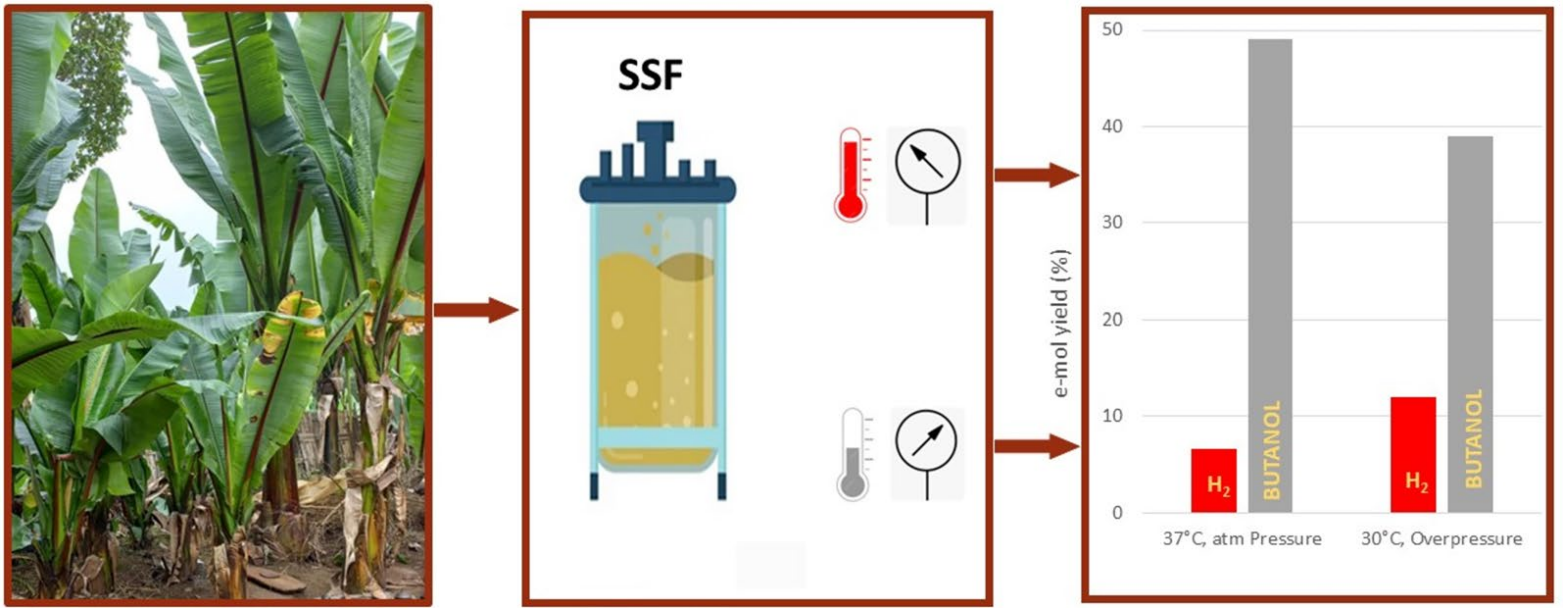

**Supplementary Information:**

The online version contains supplementary material available at 10.1186/s40643-024-00809-w.

## Background

The impacts of human-driven global warming, such as the melting of sea ice, rising sea levels, unpredictable heat waves, wildfires, and intense rainfall, are getting worse day by day. Although some countries have made progress in adapting their climate action plans to achieve net-zero emissions, the situation remains challenging (Rajak 2021; Michael and Maria 2023). According to the International Energy Agency (IEA) report (2022), CO_2_ emissions increased by 6% from 2020 to 2021, the highest emission level recorded for a single year. This increase in emissions clearly indicates that the world is far from achieving the goal of limiting warming to 1.5 °C, as set by the United Nations (Michael and Maria 2023; International Energy Agency (IEA) 2022). The goal can be achieved by shifting global energy consumption from fossil fuels to biofuels such as hydrogen and butanol, which can potentially reduce carbon emissions and establish a more sustainable energy system (Midhun Prasad et al. 2023).

In recent years, hydrogen has become increasingly popular as an energy carrier for fuelling vehicles, generating electricity, and various industrial applications (Yadav et al. 2021; Goria et al. 2023). The high energy content of hydrogen (140 MJ/kg) and the fact that its combustion produces only water as a byproduct make it an ideal clean fuel for various applications (Singh et al. 2020). Similarly, butanol is a highly versatile fuel that can be blended with gasoline in high proportions, used in existing engines, and easily transported through current pipeline infrastructure. This versatility arises from its excellent properties, such as higher energy density (29 MJ/L), lower vapor pressure (0.53 kPa), lower hygroscopicity, and lower volatility than ethanol (Rajagopalan et al. 2016; Sarkar and Yaser 2014). Several studies have shown that biological processes can successfully produce butanol and hydrogen. However, there are still numerous challenges that need to be addressed to make this process competitive with other processes. These includes substrate availability (Valles et al. 2020), low production yield (Yadav et al. 2021; Kumar et al. 2012), scalability, high processing cost (Zheng et al. 2015; Cheng et al. 2011), and maintaining optimal fermentation conditions (Wu et al. 2021; Chen et al. 2014).

Lignocellulosic biomass is readily available and economically viable worldwide and can be obtained from agricultural residues, industrial wastes, forest residues, and municipal wastes (Valles et al. 2020). It is composed of cellulose, hemicellulose, and lignin, which are the three components of plant cell walls. These components can be broken down using enzymes and other chemicals, allowing subsequent utilisation by microorganism as a substrate for biofuel production, such as hydrogen and butanol (Dong et al. 2016). Enset fiber could be a promising feedstock for biofuel production. It is an Ethiopian agricultural residue obtained from the Enset plant. A large amount of Enset fiber goes to waste after the traditional Enset food processing in Ethiopia, which feeds more than 20 million people (Borrell et al. 2019). Enset fiber contains 67.1% cellulose, 15.6% hemicellulose, and 5.1% lignin (Seid et al. 2022). A recent study conducted by Seid et al. (2022) examined the potential of Enset fiber for butanol production using the separate hydrolysis and fermentation (SHF) method, comparing it with various other Enset plant residues. The results revealed that Enset fiber holds significant promise as a viable resource for butanol production and has a higher hydrolysis conversion efficiency than other Enset plant residues. However, the main challenges for the SHF process were substrate inhibition, low butanol yield and productivity, the complexity of operational procedures requiring multiple distinct steps, and the economic viability of the process (Fatehi 2013; Guo et al. 2022).

Several studies showed that butanol can be produced by combining pretreated biomass, enzymes, and *Clostridia* species in a single bioreactor. This process is called simultaneous saccharification and fermentation (SSF), enables the simultaneous occurrence of sugar release and fermentation (Valles et al. 2020; Guan et al. 2016; Qi et al. 2019). Compared with the SHF process, SSF has many advantages, including minimising substrate inhibition, reducing operating costs, the overall risk of contamination, and maximising butanol productivity and yield (Salleh et al. 2019). However, the SSF process faces several challenges that limit its industrial application. These include the different optimal operating conditions for the saccharification process and acetone-butanol-ethanol (ABE) fermentation, which depend on the type of substrate and microorganism used. Therefore, finding a balance between the two processes is challenging and may affect the efficiency and productivity of butanol production (Cheng et al. 2019; Md Razali et al. 2018).

ABE fermentation consists of two distinct phases: acidogenic and solventogenic phases. During the acetogenic phase, the strain undergoing exponential growth produces hydrogen, as well as acids such as acetic acid and butyric acid. Due to the accumulation of these acids, cell growth begins to slow down, and the metabolism of the strain shifts into the solventogenic phase. In this phase, the strain produces solvents such as acetone, butanol, and ethanol as a survival mechanism (Yadav et al. 2021; Singh et al. 2019). *Clostridium saccharoperbutylacetonicum* is well known for producing butanol and hydrogen as a sugar fermentation product, making it ideal for biofuel production (Alalayah et al. 2008). The strain is versatile, capable of using a variety of substrates, resistant to solvents, and can thrive in harsh environments (Yao et al. 2017). Studies showed that several factors, including temperature, pH, electron flow and reducing equivalents, play a significant role in *C. saccharoperbutylacetonicum* fermentation, influencing the amount of hydrogen, -butanol, acetone and ethanol (Alalayah et al. 2008; Wu et al. 2017; Nakayama et al. 2013; Yerushalmi et al. 1985). These factors contribute to a competitive relationship between the two products. Therefore, optimal fermentation conditions are crucial for maximising both hydrogen and butanol production. This study aimed to maximize hydrogen and butanol production from Enset fiber by optimizing the SSF process. In addition, we examined the impact of controlling pH on the SSF process from Enset fiber. Furthermore, we attempted to maximize butanol productivity through the PSSF process at a high substrate loading with Enset fiber. It is worth noting that this report is the first study on the simultaneous production of hydrogen and butanol from Enset fiber using the SSF process.

## Results

### Effect of SSF process parameters on butanol production

This work investigates process parameters to improve butanol production from Enset fiber using the SSF process. Important parameters, such as agitation speed, cellulase loading and substrate loading were selected and optimized. The preliminary results showed that *C. saccharoperbutylacetonicum* fermentation produced metabolites only between temperatures of 28 °C and 37 °C; however, no metabolites were detected above 37 °C. Unless otherwise stated, all subsequent experiments were carried out at a constant temperature of 30 °C and an initial pH of 6.8. Table 1 shows the effect of agitation speed on butanol production in the SSF process and glucose production in the saccharification process (control experiment) from Enset fiber. Statistical analysis with p < 0.05 showed a significant difference in butanol concentration between different agitation speeds in the SSF process (see Additional file 1: Table S1). After 72 h of the SSF process, a maximum butanol concentration of 11.48 g/L was produced at an agitation speed of 100 rpm compared to other fermentations. At this speed, the strain consumed almost all glucose*,* resulting in a butanol yield of 0.23 g/g and productivity of 0.16 g/(L h). However, at 130 rpm, butanol production was lower (10.33 g/L), and 9.48 g/L glucose remained unconsumed. On the other hand, the saccharification process showed that the enzyme released more glucose (44.57 g/L) at 130 rpm than at other speeds after 72 h.

**Table 1:** Effect of agitation speed on butanol production in the SSF process and glucose production in the saccharification process in bottles^a,b^

Agitation speed (rpm)	SSF process				Saccharification process	
	Final glucose concentration (g/L)	Butanol concentration (g/L)	Butanol yield (g/g)	Butanol productivity (g/(L h))	Glucose released (g/L)	Maximum glucose production rate (g/(L h))
0	7.93 ± 1.12	9.64 ± 0.97	0.19	0.13	37.13 ± 0.14	2.93
100	0.24 ± 0.17	11.48 ± 0.22	0.23	0.16	38.94 ± 1.99	3.29
130	9.48 ± 1.36	10.33 ± 0.08	0.21	0.14	44.57 ± 1.01	2.96

^a^All calculations accounted for 2.5 g Enset fiber in 50 ml medium at 72 h fermentation period

^b^Values are means from triplicate bottles

In addition, the effects of cellulase loading on butanol production from Enset fiber were investigated. Table 2 presents the effects of different cellulase loadings on butanol production in the SSF process and glucose production in the saccharification process from Enset fiber. The result showed no significant difference in butanol production in the SSF process between 16 and 24 FPU/g, while 9 FPU/g cellulase loading resulted in a slightly lower butanol production of 10.73 g/L. The saccharification process released a higher glucose concentration of 47.47 g/L at 30 FPU/g cellulase loading, but the SSF process showed the lowest butanol concentration of 9.6 g/L and a yield of 0.19 g/g.

**Table 2:** Effect of cellulase loading on butanol production in the SSF process and glucose production in the saccharification process in bottles^a,b^

Cellulase loading (FPU/g)	SSF process				Saccharification process	
	Final glucose concentration (g/L)	Butanol concentration (g/L)	Butanol yield (g/g)	Butanol productivity (g/(L h))	Glucose released (g/L)	Maximum glucose production rate (g/(L h))
9	3.73 ± 0.34	10.73 ± 0.29	0.21	0.15	29.67 ± 0.33	1.11
16	4.99 ± 0.18	11.23 ± 1.28	0.22	0.16	39.47 ± 0.62	1.85
24	0.24 ± 0.19	11.48 ± 0.22	0.23	0.16	38.94 ± 1.99	3.29
30	6.82 ± 1.42	9.60 ± 0.59	0.19	0.13	47.47 ± 0.75	3.21

^a^All calculations accounted for 2.5 g Enset fiber in 50 ml medium at 72 h fermentation period

^b^Values are means from triplicate bottles

The effects of substrate loading with Enset fiber on butanol production in the SSF process were also examined. Table 3 shows how substrate loading affects butanol production in the SSF process and glucose production in the saccharification process from Enset fiber. The results showed that the same butanol concentration of 11 g/L was observed at both 5 and 7% (w/v) substrate loading of Enset fiber. However, at 7% (w/v) substrate loading, the butanol yield was much lower (0.16 g/g), and 12.42 g/L of glucose remained unfermented compared to other substrate loadings. On the other hand, higher butanol yields were achieved at 2 and 3% (w/v) substrate loadings despite lower butanol concentration and productivity. In the saccharification process, the highest glucose concentration (44.74 g/L) was released at a substrate loading of 7% (w/v), but the glucose production rate was slower than other substrate loadings.

**Table 3:** Effect of substrate loading on biobutanol production in the SSF process and glucose production in the saccharification process in bottles^a,b^

Substrate loading (% (w/v)	SSF process				Saccharification process	
	Final glucose concentration (g/L)	Butanol concentration (g/L)	Butanol yield (g/g)	Butanol productivity (g/(L h))	Glucose released (g/L)	Maximum glucose production rate (g/(L h))
2	0.01 ± 0.00	5.23 ± 0.14	0.26	0.07	16.02 ± 1.05	1.87
3	0.01 ± 0.00	7.96 ± 0.17	0.27	0.11	25.44 ± 1.54	1.83
5	4.99 ± 0.18	11.23 ± 1.28	0.22	0.16	39.47 ± 0.62	1.85
7	12.42 ± 0.43	11.06 ± 0.09	0.16	0.15	44.74 ± 1.78	1.64

^a^All calculations accounted for each mass of Enset fiber in 50 ml medium at 72 h fermentation period

^b^Values are means from triplicate bottles

**Fig. 1:**
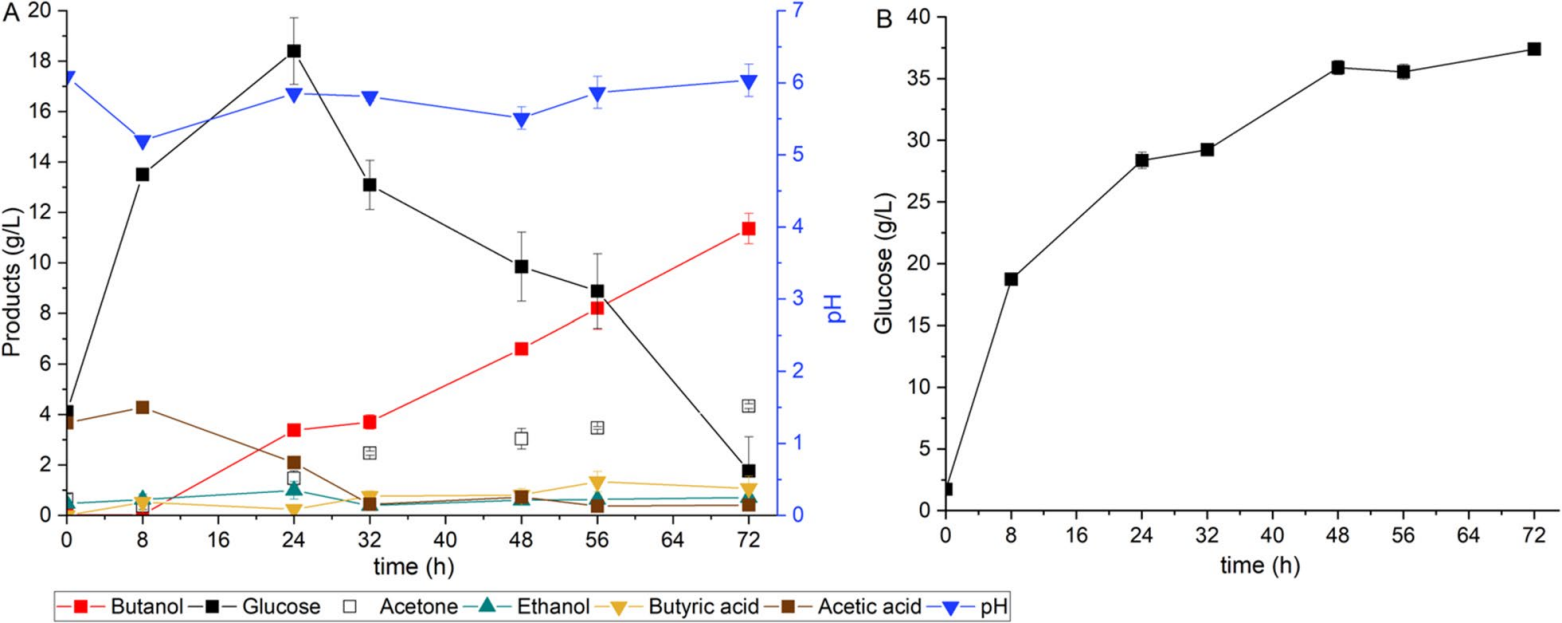
Validation experiment at 5% substrate loading, 16 FPU/g cellulase loading, and 100 rpm **A** SSF process, **B** saccharification process (control experiment). All values are means from triplicate bottles

Overall, at the end of fermentation, the validation experiment confirmed that under the optimum process parameters, including 5% (w/v) substrate loading, 16 FPU/g cellulase loading, and 100 rpm agitation speed, the SSF process from Enset fiber resulted in a maximum butanol concentration of 11.36 g/L, with a corresponding yield of 0.23 g/g and a productivity of 0.16 g/(L h). In addition, 4.33 g/L acetone and 0.71 g/L ethanol were produced, while 1.77 g/L glucose, 0.41 g/L acetic acid, and 1.08 g/L butyric acid remained unconsumed (Fig. 1A). The validation experiment in the saccharification process also showed an almost similar glucose concentration (37.4 g/L) after 72 h. However, the maximum glucose production rate of this experiment was slightly faster than the previous one at 2.34 g/(L h) (Fig. 1B).

### The impact of controlled and uncontrolled pH values in the SSF process on butanol production

This study aimed to establish a small-scale SSF process for butanol production from Enset fiber in a 2.5 L bioreactor and to investigate the influence of pH control on the process. All fermentations were carried out under the optimal conditions of 5% (w/v) substrate loading, 16 FPU/g cellulase loading, a temperature of 30 °C, and an initial pH of 6.8, as determined from the previous bottle experiments, except for the stirrer speed, which was 150 rpm. The SSF process was scaled up in a 2.5 L stirred tank reactor with a pressure relief valve that maintained the hydrogen-rich gas in the medium. The bioreactor pressure reached an absolute pressure of 1.55 bar in about 5 h; after that, the valve opened and regulated the pressure, while the excess gas was released through BlueVCount and collected in a gas bag. The pressure dropped to 1.52 bar after 24 h of fermentation and remained constant until 54 h. Then, the pressure fluctuated between 1.23 and 1.48 bar until the end of fermentation.

**Fig. 2:**
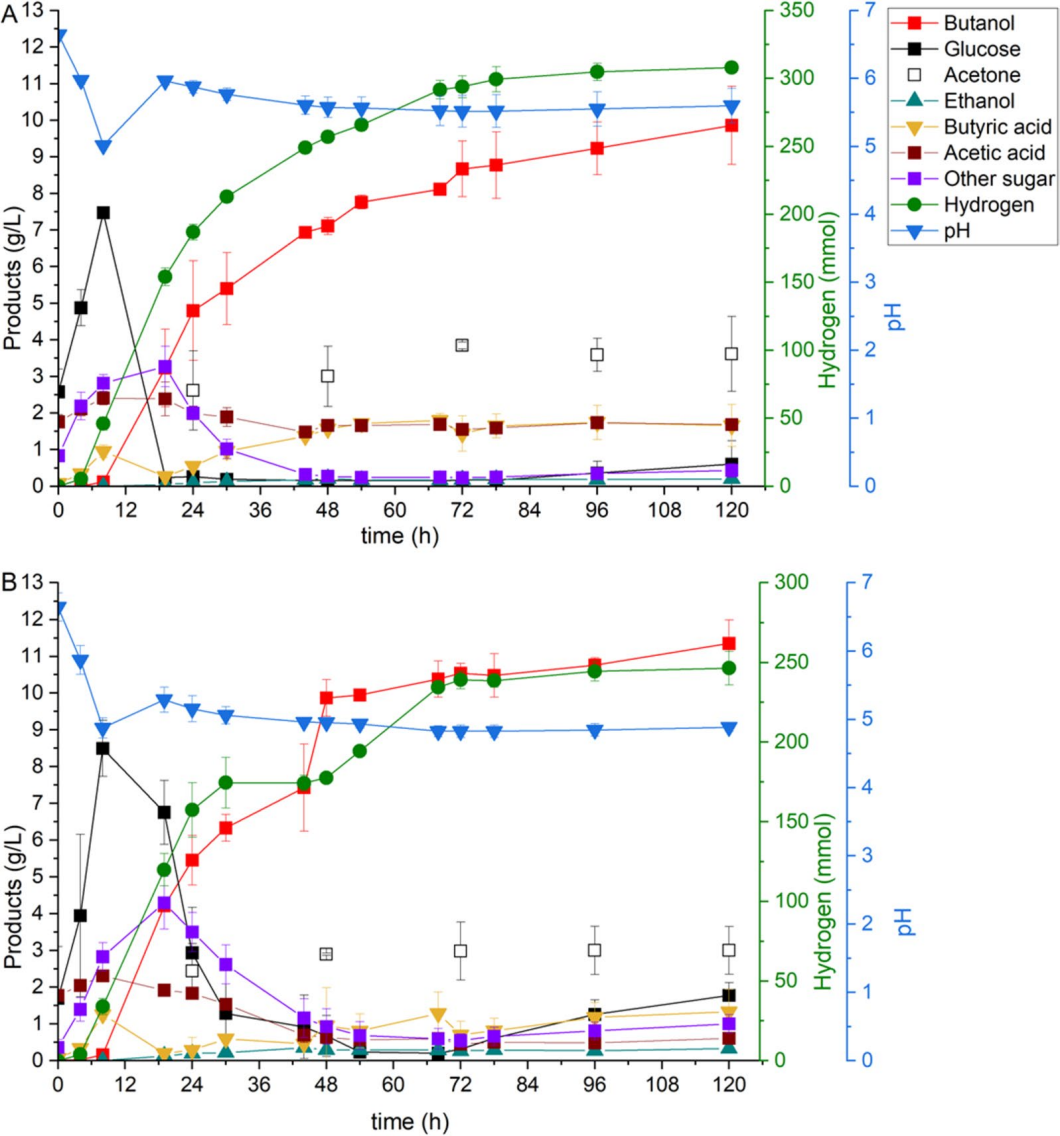
Metabolites in the SSF process from Enset fiber with **A** pH-controlled and **B** pH-uncontrolled. All values are means from duplicate fermentations in the bioreactor

Previous studies have shown that maintaining a pH of above 5.0 during ABE fermentation enhances butanol production (Yang et al. 2013; Feng et al. 2020), and the optimal pH range for cellulase enzyme activity during saccharification lies between 5.0 and 5.5 (Balsan et al. 2012). In this study, the influence of pH control (above 5.0) on butanol production in the SSF process was examined using the same bioreactor setup, and the results were compared with those of pH-uncontrolled fermentation. Figure 2A, B shows the metabolites and pH profiles in the SSF process from Enset fiber with the pH-controlled and pH-uncontrolled fermentations. The pH value in both fermentations decreased from 6.8 to 5.0 within 8 h of inoculation. In the pH-controlled fermentation, the base pump was turned on after 8 h to keep the pH above 5.0; however, after 19 h, no addition of NaOH was observed. Within 19 h, the pH of this fermentation reached a peak pH of 5.96 and then decreased to 5.53 after 68 h. Afterwards, it fluctuated between 5.51 and 5.60. In contrast, the pH-uncontrolled fermentation showed a slight increase in pH to 5.29 after 19 h and a gradual decrease to 4.83 after 68 h, where it remained constant until the end of the fermentation.

In addition, a comparison was made between the pH-controlled and pH-uncontrolled SSF processes regarding their effects on liquid metabolites. In both fermentations, as shown in Fig. 2A, B, the enzyme released a maximum glucose concentration of about 8 g/L after 8 h, which was similar to the time when the pH was lowered to 5.0. Following that, a higher glucose concentration was observed in the pH-uncontrolled fermentation until the end of the fermentation than in the pH-controlled fermentation. Similarly, other sugars such as xylose, mannose and galactose were detected in higher concentrations in the pH-uncontrolled fermentation than in pH-controlled. In both fermentations, the strain began producing butanol around 19 h. However, the pH-uncontrolled fermentation had a faster production rate of 0.23 g/(L h) than the pH-controlled one. It also had a higher butanol concentration of 10.54 g/L after 72 h compared to 8.67 g/L in the pH-controlled fermentation. At the end of fermentation, butanol concentration was increased to 11.35 g/L, yielding 0.23 g/g in the pH-uncontrolled fermentation, however the productivity decreased to 0.095 g/(L h) compared to a fermentation time of 72 h. Likewise, in the pH-controlled fermentation, the butanol concentration increased to 9.86 g/L, yielding 0.20 g/g and productivity of 0.082 g/(L h). In addition, both fermentations produced other solvents, such as acetone and ethanol. However, the difference in acetone production between the pH-controlled and pH-uncontrolled fermentations was insignificant at 3.61 g/L and 3.00 g/L, respectively, and ethanol was detected in small amounts in both fermentation, which was lower than 0.5 g/L. The acid compositions, such as acetic acid and butyric acid, also differed in both fermentations. Acetic acid and butyric acid levels were higher in the pH-controlled fermentation than in the pH-uncontrolled fermentation. As with butanol production, hydrogen production showed the opposite trend when comparing pH-controlled and pH-uncontrolled fermentations. A higher amount of hydrogen was observed in the pH-controlled fermentation at 308.02 mmol than in the pH-uncontrolled fermentation at 246.47 mmol.

### PSSF process at high substrate loading with Enset fiber

**Fig. 3:**
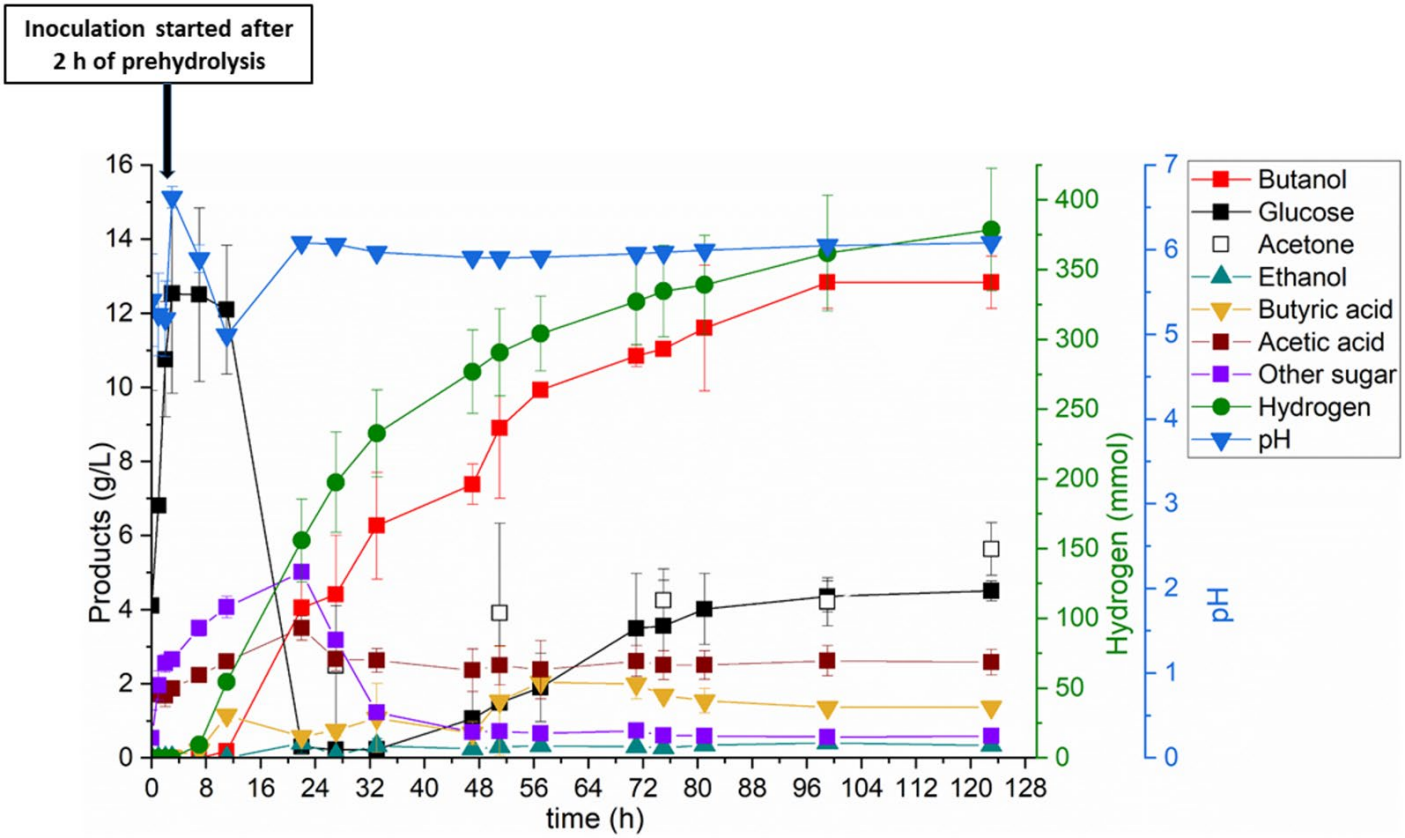
Metabolites in the PSSF process at high substrate loading with Enset fiber. All values are means from duplicate fermentations in the bioreactor

The purpose of this study was to maximize butanol productivity using the PSSF process at high substrate loading with Enset fiber. This experiment was done in a bioreactor with pH-controlled fermentation similar to the experiment described above. Figure 3 shows the metabolites produced in the PSSF process at a substrate loading of 7% (w/v) using Enset fiber as a carbon source. The prehydrolysis step lasted for 2 h, followed by a 1 h transition period to adjust the temperature and initial pH value, and the enzyme hydrolysed the Enset fiber into 12.53 g/L of glucose and 2.66 g/L of other sugars, such as xylose, mannose, and galactose. In addition, we observed that the Enset fiber was partially degraded, and the medium was homogenized at the end of this process. After inoculation with *C. saccharoperbutylacetonicum*, the PSSF process achieved a faster butanol production rate of 0.21 g/(L h) than the pH-controlled SSF process but a slightly similar rate to the pH-uncontrolled SSF process. However, the butanol concentration after 72 h was higher at 11.04 g/L in the PSSF process than in the SSF process in both pH-controlled and uncontrolled fermentation. At the end of fermentation, the butanol concentration and productivity in the PSSF process were increased to 12.84 g/L and 0.104 g/(L h), respectively. Although the butanol concentration and productivity were higher in the PSSF process than in the SSF process, the butanol yield of 0.18 (g/g) was significantly lower than in the SSF process. Furthermore, the PSSF process produced a higher concentration of acetone (5.64 g/L) and a higher amount of hydrogen (378.69 mmol) than the SSF process. However, at the end of the fermentation, we found that 4.51 g/L glucose, 2.58 g/L acetic acid and 1.37 g/L butyric acid remained unused.

### Effect of SSF process parameter on hydrogen production

**Fig. 4:**
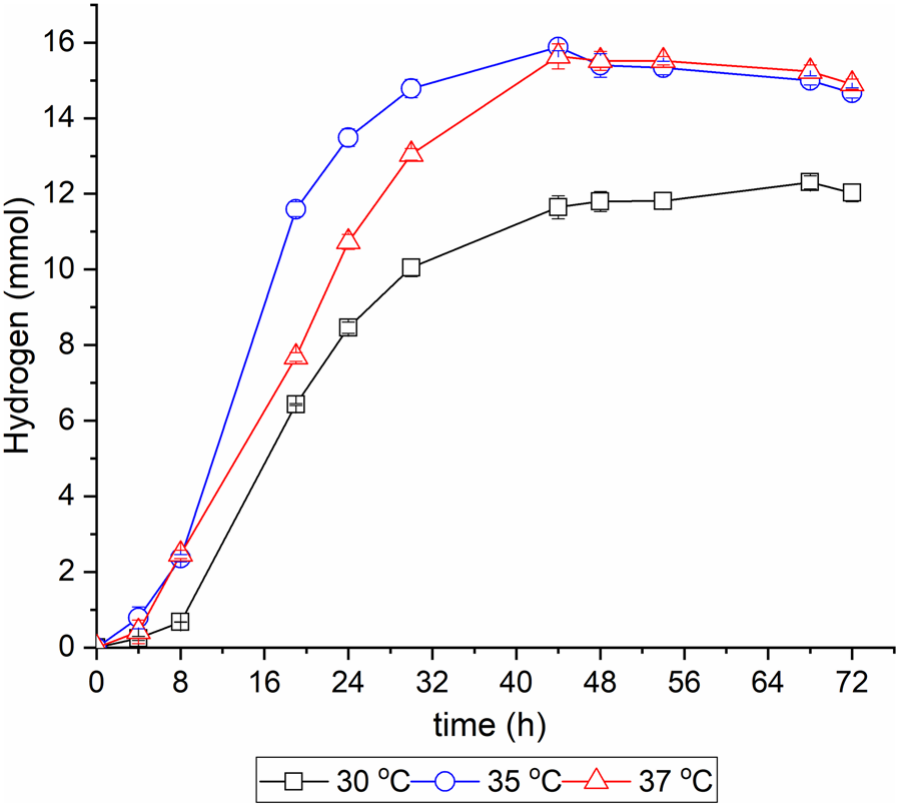
Effect of temperature on hydrogen production in the SSF process. All values are means from triplicate bottles

The objective of this study was to examine the influence of SSF process parameters on hydrogen production from Enset fiber. The key process parameters affecting hydrogen production were selected and optimized, including temperature, initial pH, and hydrogen partial pressure. At the same time, other factors were kept constants for all experiments based on results from previous bottle experiments. Figure 4 shows the effect of temperature on hydrogen production from Enset fiber in the SSF process. After 44 h of incubation, at both 35 °C and 37 °C, hydrogen production reached a maximum of 15.75 mmol and then slightly declined at both temperatures. At the end of fermentation, hydrogen production was lower at 30 °C (12.03 mmol) than at other temperatures. However, the butanol yield and productivity were much higher at 30 °C, with 0.23 g/g and 0.17 g/(L h), respectively. In contrast, at 37 °C, the butanol yield was lower at 0.17 g/g, while the hydrogen yield was higher at 133.50 mL/g-Enset fiber compared to others (see Additional File 1: Table S2).

**Fig. 5:**
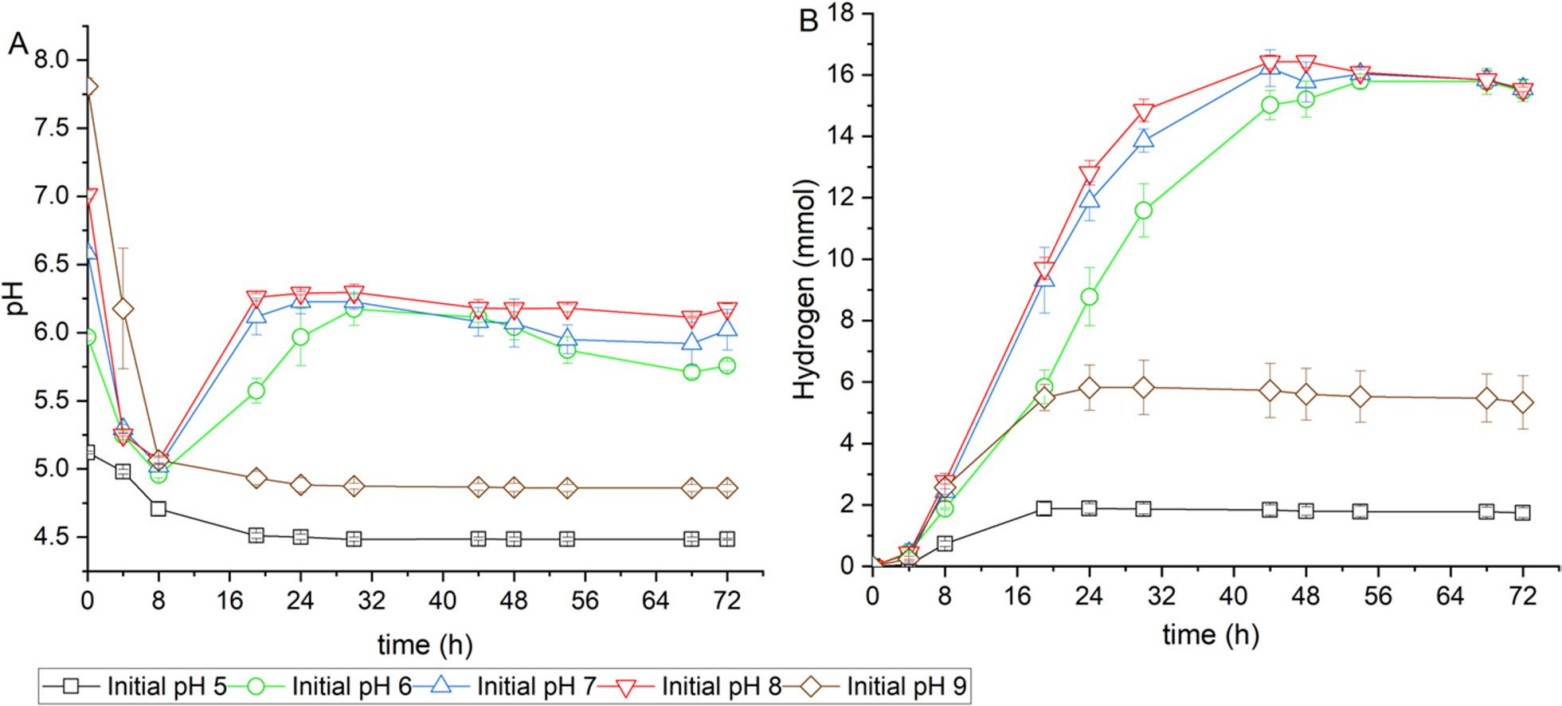
Effect of initial pH values on hydrogen production in the SSF process from Enset fiber **A** pH profile and **B** hydrogen production. All values are means from triplicate bottles

In addition, the effects of initial pH value on hydrogen production from Enset fiber were investigated. The initial pH of the medium was adjusted to 5.0, 6.0, 7.0, 8.0, and 9.0 at the beginning of the experiment. However, immediately after inoculation, the pH changed to 5.12, 5.97, 6.58, 7.01, and 7.81, respectively, due to the low pH value of the preculture. Figure 5A shows the pH changes during the SSF process with Enset fiber at different initial pH values. The initial pH of 6.0, 7.0, and 8.0 showed similar pH trends, decreasing at 8 h, and increasing to about 6.23 after 24 h. At the end of fermentation, they had nearly the same pH of 6.01, except for the initial pH of 6.0, which was slightly lower at 5.76. In contrast, the initial pH of 5.0 and 9.0 showed similar pH trends, dropping below 5.0 after 8 h and remaining low until the end of fermentation. Similarly, the initial pH values affected the amount of hydrogen produced by the SSF process, as shown in Fig. 5B. After 44 h of fermentation, the highest hydrogen production was observed at 16.32 mmol at initial pH values of 7.0 and 8.0. However, the initial pH of 8.0 had a slightly faster rate of 0.53 mmol/h than the others. At the end of fermentation, a similar hydrogen yield (139 mL/g-Enset fiber) was observed at all initial pH values of 6.0, 7.0 and 8.0. The lowest amounts of hydrogen were found at both initial pH of 5.0 and 9.0, with 1.74 mmol and 5.33 mmol, respectively. Similar to the amount of hydrogen, there was no significant difference in butanol yield (0.18 g/g-Enset fiber) between the initial pH of 6.0, 7.0, and 8.0 at the end of fermentation. In contrast, at the end of fermentation, the lowest butanol yield was observed at initial pH of 5.0 and 9.0; however, a significantly higher glucose concentration of 38 g/L and 30 g/L were observed at an initial pH of 5.0 and 9.0, respectively (see Additional File 1: Table S3).

**Fig. 6:**
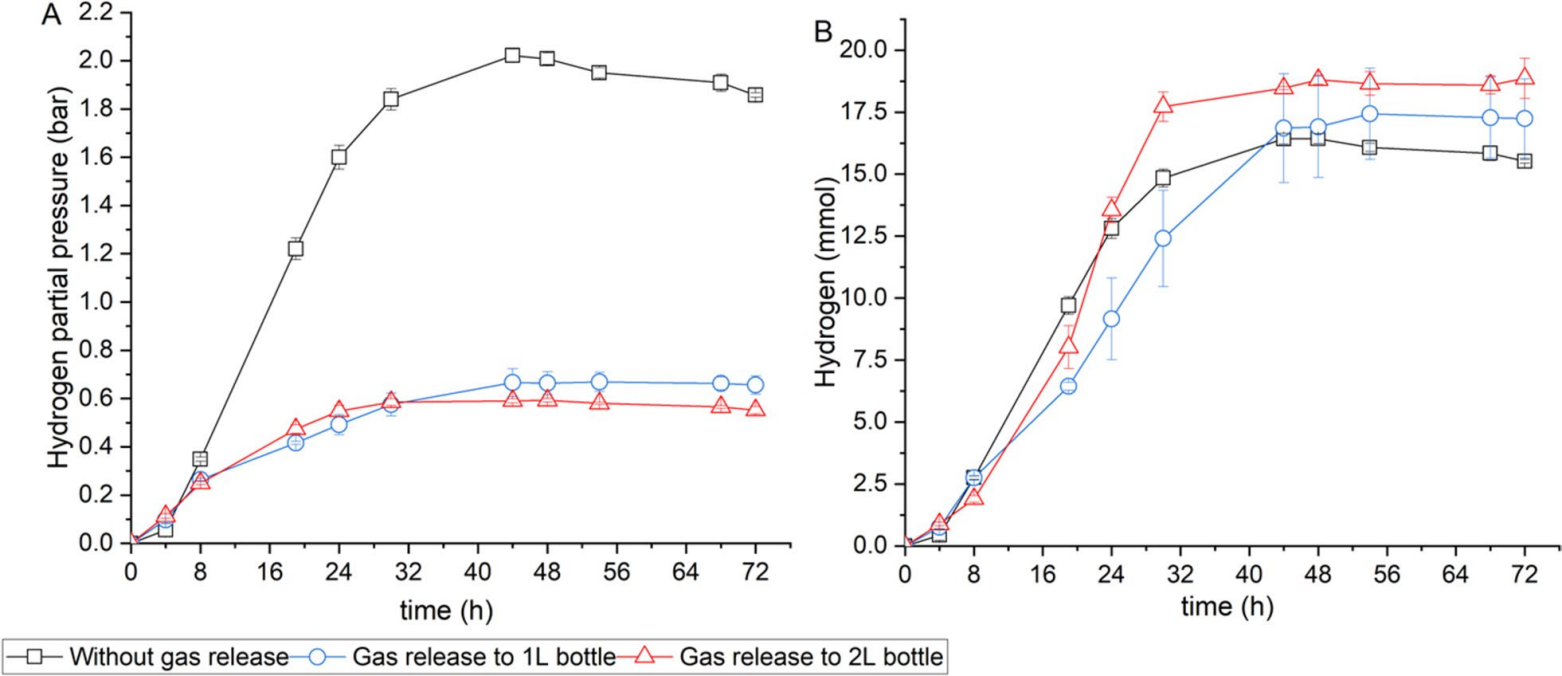
Effect of gas release strategy in the SSF process from Enset fiber **A** hydrogen partial pressure **B** hydrogen production. All values are means from triplicate bottles

The effects of hydrogen partial pressure on hydrogen production in the SSF process from Enset fiber were also investigated. Figure 6A, B shows the hydrogen partial pressure and hydrogen production in the SSF process from Enset fiber under different gas release strategies. Statistical analysis with p < 0.05 indicated a significant difference in hydrogen production between different hydrogen partial pressure in the SSF process (see Additional file 1: Table S4). In both experiments, where gas was released into 1-L and 2-L bottles, the hydrogen partial pressure was lower compared to the control experiment without gas released. However, the gas released into the 1-L bottle resulted in a slightly lower hydrogen partial pressure than that released into the 2-L bottle (Fig. 6A). Similarly, at the end of fermentation, there was a slight difference in hydrogen production of 17.24 mmol and 18.86 mmol between the fermentation, where the gas was released into 1-L and 2-L bottles, respectively. However, a smaller amount of hydrogen (15.52 mmol) was observed in the control experiment (Fig. 6B). Furthermore, it was found that there was little difference in butanol production between all experiments (see Additional File 1: Table S5). Overall, at the optimum process parameters, including a temperature of 37 °C, an initial pH of 8.0, and the lowest hydrogen partial pressure, a maximum hydrogen yield of 168.99 mL/g-Enset fiber was achieved in the SSF process from Enset fiber.

### Hydrogen production in the SSF process at atmospheric pressure

**Fig. 7:**
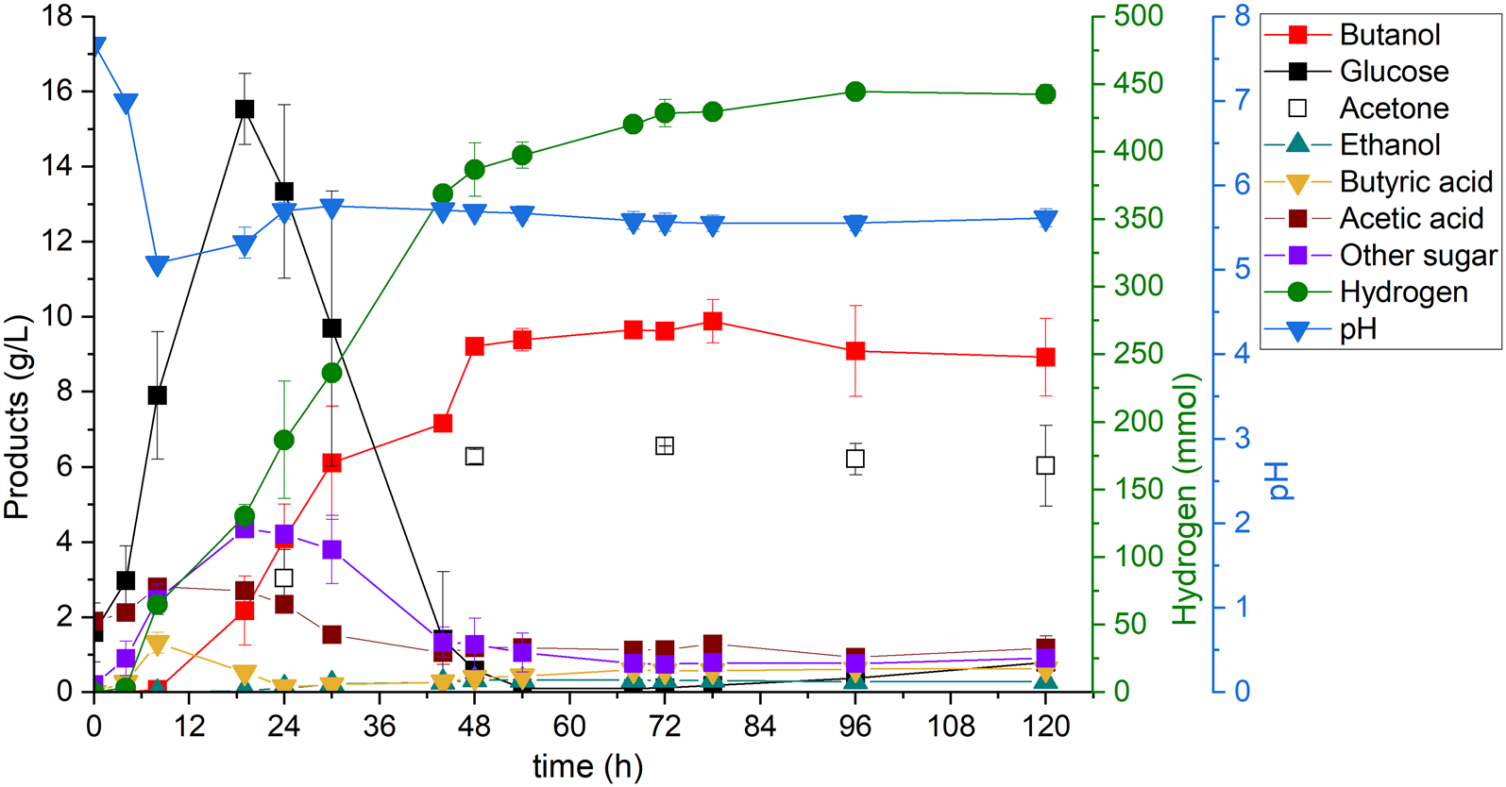
Metabolites in the SSF process from the Enset fiber SSF process at atmospheric pressure. All values are means from duplicate fermentations in the bioreactor

This study aimed to develop a small-scale SSF process for hydrogen production from Enset fiber in a 2.5 L bioreactor. This was achieved by using optimal process parameters, including temperature (37 °C), initial pH (8.0), and atmospheric pressure as determined in bottle experiments. The SSF process was scaled up using a 2.5 L stirred tank reactor operated at atmospheric pressure and without a pH control method. Initially, the outlet tube was closed for 4 h to maintain an anaerobic environment in the bioreactor. During this period, the strain produced its gas, and the pressure was increased to an absolute pressure of 1.3 bar. Following this, the bioreactor outlet was connected to the BlueVCount, which released gases into the gas bag, thereby reducing the pressure to the atmospheric level of 1 bar. Figure 7 shows the metabolites and pH profiles in the SSF process from Enset fiber at atmospheric pressure. The result of this study was compared to a similar SSF process, except that the SSF was performed at 30 °C, with an initial pH of 6.8, without pH control and under overpressure (1.55 bar) (see Fig. 2B). Unless otherwise stated, this was the reference process for the comparison.

After inoculation, hydrogen gas production was started in 4 h in both fermentations, and the amount was similar at 3.64 mmol. In 24 h, the amount of hydrogen in the SSF process at 37 °C reached 186.70 mmol, whereas at 30 °C, it reached 157.36 mmol with a maximum hydrogen production rate of 6.56 mmol/h. Subsequently, hydrogen production in the SSF process at 37 °C further increased to 368.85 mmol within 44 h, with a maximum hydrogen production rate of 8.38 mmol/h. However, in the SSF process at 30 °C, the production rate slowed to 3.96 mmol/h and reached 174.31 mmol of hydrogen. Furthermore, after 72 h, a higher amount of hydrogen was found at 428.53 mmol in the SSF process at 37 °C than in the SSF process at 30 °C with 239.14 mmol. However, hydrogen production did not change significantly when the fermentation time was extended to 120 h, both fermentations showed a slight increase in hydrogen production with 442.57 mmol and 246.47 mmol for the SSF processes at 37 °C and 30 °C, respectively. In addition, a higher hydrogen yield of 198.27 mL/g-Enset fiber was achieved at the end of fermentation in the SSF process at 37 °C compared to the yield of 110.42 mL/g-Enset fiber at 30 °C.

Furthermore, the pH profiles and liquid metabolites of the SSF processes showed different results at different temperatures and initial pH values (Figs. 2B and 7). The pH of both fermentations decreased to 5.0 in the first 8 h of fermentation and then increased slightly above 5.0 in the next 19 h. After that, the pH value in the SSF process at 37 °C remained above 5.0 and fluctuated between 5.5 and 5.76, while the pH value in the SSF process at 30 °C dropped again for about 44 h and varied between 4.82 and 4.96. At the end of fermentation, the SSF process at 37 °C had a higher pH of 5.6 than the SSF process at 30 °C, which had a lower pH of 4.89. Similarly, the glucose concentration released by the enzyme after 8 h of fermentation was the same in both SSF processes (8.2 g/L). However, after 19 h, the SSF process at 37 °C reached a maximum glucose concentration of 15.54 g/L, while the SSF process at 30 °C did not increase further. At the end of fermentation, the strain consumed almost all of the glucose in the SSF process at 37 °C, while 1.77 g/L glucose remained unused at 30 °C. Moreover, both SSF processes had a maximum of 4.25 g/L of other sugars, such as xylose, mannose and galactose, which were also consumed by the end of fermentation. In addition, at the end of fermentation, the liquid metabolites such as butanol and acetone differed depending on the temperature and initial pH value. The SSF process at 30 °C produced more butanol (11.35 g/L) than the SSF process at 37 °C (8.92 g/L). However, the opposite was observed for acetone, which was higher in the SSF process at 37 °C (6.04 g/L) than the SSF process at 30 °C (3.00 g/L). Also, the SSF process at 37 °C contained more acetic acid and less butyric acid than the SSF process at 30 °C.

Additionally, the electron and carbon balance calculations for both fermentations were estimated (see Additional File 1: Table S6 & Table S7). When operated at 37 °C and atmospheric pressure, the process achieved a yield of 12% e-mol of hydrogen and 38% e-mol of butanol. In contrast, at 30 °C and an overpressure of 0.55 bar, the e-mol yields were significantly different at 6% for hydrogen and 48% for butanol. This demonstrates the sensitivity of the process to variations in operating conditions. In addition, 75–78% of carbon was recovered as metabolites in both fermentations. The remaining carbon in the Enset fiber might not be degraded by enzymes, or it could be carbon present in the *Clostridial* cell biomass.

## Discussions

### Maximizing butanol production from Enset fiber using the SSF process

- One of the primary challenges in maximizing butanol production in the SSF process using *C. saccharoperbutylacetonicum* is the large temperature difference required for ABE fermentation and saccharification, typically between 30 and 50 °C (Olofsson et al. 2008). The cellulase enzymes require higher temperatures of 40–50 °C to efficiently degrade lignocellulosic biomass (Balsan et al. 2012), while the strain prefers lower temperatures of 30 °C for optimal butanol production (Yao et al. 2017).. In this study, we observed that *C. saccharoperbutylacetonicum* was able to grow and produce butanol from pretreated Enset fiber using the SSF process within a temperature range of 28–37 °C, and the maximum butanol production was observed at 30 °C. This could be due to the structural properties of Enset fiber, which has a high cellulose content (67.1%) and a low lignin content (5.1%), making it a promising feedstock for the SSF process (Seid et al. 2022). Lignin is a natural polymer that acts as a barrier to prevent the enzymatic hydrolysis of cellulose and hemicellulose. It can be degraded by various pretreatment methods such as dilute acid, dilute alkali, steam explosion, and liquid hot water; however, depending on the biomass and the specific type of pretreatment, the pretreatment methods can lead to lignin degradation. The resulting lignin degradation products, such as aromatic alcohols, phenolic compounds, furan derivatives, and organic acids, act as soluble inhibitors that can affect enzyme activity and strain growth (Öhgren et al. 2007; Santos et al. 2019). Previous studies have shown that the SSF process is affected by inhibitory compounds resulting from the degradation of lignin in alkali-pretreated switchgrass (Guan et al. 2016) and acid-pretreated corn stover (Qureshi et al. 2014), which inhibits the *Clostridial* strain. However, alkali-pretreated Enset fiber did not generate any inhibitory compounds, according to a study (Seid et al. 2022).

In this study, the effects of other process parameters, such as agitation speed, cellulase loading and substrate loading on the SSF process were further optimized while maintaining a constant temperature (30 °C) and initial pH (6.8), which were found to be the optimal parameters for ABE fermentation using *C. saccharoperbutylacetonicum* (Yao et al. 2017). Mixing the SSF medium improves the mass transfer between biomass, enzyme, microorganism, and nutrients in the SSF process. However, agitation speed affects the ABE fermentation and saccharification process differently. The bottle experiments in the current study revealed that the SSF process produced the highest butanol concentration at an agitation speed of 100 rpm. However, higher rotation reduced the butanol concentration by 10% in the SSF process. This reduction might be due to excessive mixing, which can cause hydrogen gas to escape from the liquid medium to the gas phase, potentially impacting butanol production. On the other hand, the saccharification process (control experiment) benefited from high rotation; more glucose was produced at higher agitation speeds, possibly due to the enhanced interaction between the enzyme and the Enset fiber. The results of this study were similar to those of Al-Shorgani N. et al. (2015), who investigated the effects of agitation on butanol production in ABE fermentation using the same strain as in this study. They found that 100 rpm was the optimal agitation speed for maximum butanol production, and at higher speeds, butanol production decreased.

Maintaining an optimal cellulase loading during the SSF process is essential for maximizing the butanol production and ensuring the overall cost-effectiveness of the process. In this study, it was observed that there was only a 4% difference in butanol yield between the cellulase loadings of 16 and 24 FPU/g, despite a 33% increase in the cellulase loading. For this reason, a cellulase loading of 16 FPU/g was selected as an optimal point for the SSF process from Enset fiber. Study shows that enzymes contribute to 40% of the total cost of biofuel production from lignocellulosic biomass (Kumar Ramamoorthy et al. 2019) and reducing cellulase loading is economically beneficial when the yield loss is less than 6–7% (Olofsson et al. 2008). Further increasing cellulase loading to 30 FPU/g in the SSF process resulted in a 16% decrease in butanol production. This could be due to the inhibitory effect of excessive cellulase on the strain (Oberoi et al. 2011). Compared to the previous study, this result was similar to the research conducted by Md Razali et al. (2018), which investigated the effect of cellulase loading in the SSF process using the *C. acetobutylicum* strain and reported a reduction in butanol production from pretreated oil palm empty fruit bunch as the cellulase loading increased from 20 to 30 FPU/g.

One of the challenges in butanol production from lignocellulosic biomass using the SSF process is balancing the required fermentable sugars and substrate loading, which can lead to mass transfer limitations. This study revealed that even at low substrate loading of 2% and 3% (w/v) Enset fiber, the SSF process achieved a maximum butanol yield of 0.26 (g/g) and 0.27 (g/g), respectively. In addition, 80–84% of Enset fiber was converted into glucose during the saccharification process. These proved that the SSF and saccharification processes using Enset fiber were efficient, despite the reduced enzyme activity due to the low process temperature. However, the butanol productivity was significantly lower at low substrate loading than in fermentations with high substrate loading. Butanol productivity was maximized by 45% when the substrate loading increased from 3 to 5% (w/v) of Enset fiber. However, further increases in substrate loading to 7% (w/v) showed no significant difference; instead, the butanol yield decreased by 27%, and 12.42 g/L glucose remained unconsumed. This might be due to poor mixing caused by high solid and low water content, leading to a mass transfer problem in the SSF process (Dong et al. 2016). A similar challenge was identified in a study by Guan W. et al. (Guan et al. 2016), which focused on butanol production from kraft paper mill sludge using the SSF process; they found that the slurry became too viscous, limiting mass transfer at substrate loadings of 6.3% and higher. Several studies suggest that the PSSF process can solve the mixing problem during the initial stages of fermentation in the SSF process (Wu et al. 2021; Dong et al. 2016; He et al. 2017).

Table 4 compares the butanol production from different biomass and *Clostridial* strains using the SSF process in bottle experiments. Compared with other studies, Enset fiber had the highest butanol concentration (11.36 g/L), yield (0.23 g/g), and productivity (0.16 g/(L h)) among rice straw, oil palm empty fruit bunch, and avicel at optimal process parameters. However, wheat straw had a higher butanol concentration (12.64 g/L) than Enset fiber, as reported by Qi et al. (2019), while Enset fiber had a higher butanol yield and productivity. To our knowledge, no studies have yet reported butanol production in the SSF process using *C. saccharoperbutylacetonicum*. Compared to the SHF method described in our previous study (Seid et al. 2022), which used the same strain and biomass with separate fermentation and saccharification steps, the butanol concentration was 9.9 g/L, which was lower than the current study. Furthermore, the SSF process enhanced butanol yield by 20% and productivity by 21% compared to the SHF process. This could be because the SSF process can avoid substrate inhibition by releasing the sugar gradually rather than all at once, as in the SHF process.

**Table 4:** Comparison of butanol production from different biomass using the SSF process in bottle experiments

Biomass	Pretreatment method	Strain	Process parameters	Butanol titer (g/L)	Butanol yield (g/g biomass)	Butanol productivity (g/(L h))	Refs
Enset fiber	2% (w/v) NaOH	*C. saccharo-perbutyl-acetonicum* DSM 14923	5% (w/v) substrate loading, Cellic CTec2 (16 FPU/g-substrate), initial pH = 6.8, 30 °C, 100 rpm, 72 h	11.36	0.23	0.16	This study^a^
Rice straw	Microwave-assisted hydrothermo-lysis	*C. beijerinckii* DSM 6422	9% (w/v) substrate loading, Cellic CTec2 (12 FPU/g-glucan, initial pH = 6.4, 37 °C, 150 rpm, 48 h	5.5	0.06	0.11	Valles et al. 2020)
Oil palm empty fruit bunch	2% (w/v) NaOH	*C. aceto-butylicum* ATCC 824	5% (w/v) substrate loading, acremonium cellulase (15 FPU/g-substrate), initial pH = 5.5, 35 °C, 150 rpm, 120 h	3.97	0.08	0.03	Md Razali et al. 2018)
Avicel	None	*C. aceto-butylicum* ATCC-824	5.8% (w/v) substrate loading, Cellic CTec2 (20 FPU/g-glucan), initial pH = 6.7, 36 °C, 150 rpm, 120 h	9.5	0.16	0.08	Guan et al. 2016)
Wheat straw	10% (w/v) Ammonium sulfite	*C. aceto-butylicum* ATCC 824	9% (w/v) substrate loading, Cellulase (5 FPU/g-substrate) & xylanase (10 IU/g-substrate), initial pH = 5.0, 37 °C, 150 rpm, 144 h	12.64	0.14	0.088	Qi et al. 2019)

^a^All calculations accounted for different substrate loading of Enset fiber in 50 ml medium at 72 h fermentation period

### Scaling up butanol production from Enset fiber

Obtaining comparable results in bottle and bioreactor fermentation experiments is crucial for validating scalability, optimizing conditions, achieving consistency, and gaining a comprehensive understanding of the fermentation process. This study successfully established a small-scale SSF process from pretreated Enset fiber using a 2.5 L stirred tank bioreactor by maintaining a slight overpressure inside the bioreactor. In addition, the bioreactor fermentation using the pH-uncontrolled method showed a comparable butanol production result as the bottle experiment under similar optimal process parameters, except for the stirrer speed in the bioreactor. In both experiments, there was only a small difference in butanol concentration of 7.8% after 72 h. However, after 120 h, a similar butanol concentration of 11.35 g/L was observed with a yield of 0.23 g/g. These comparable results were achieved due to the utilization of a partially similar configuration when setting up the bioreactor system, which included maintaining the hydrogen-rich gas in the bioreactor. Several studies showed that maintaining hydrogen gas during fermentation is beneficial for maximizing biobutanol production in *Clostridial* strains. Brosseau et al. (1986) observed that *C. saccharoperbutylacetonicum* requires hydrogen gas to produce butanol, and when the hydrogen gas reaches a certain level, the bacteria reduce their growth rate and start producing butanol. Similarly, in this study, the highest hydrogen production rate was observed during the initial cell growth phase of *C. saccharoperbutylacetonicum*, which remained faster until 24 h. Afterwards, the rate of hydrogen gas production slowed down, and the strain started producing butanol and entered the solventogenic phase. Furthermore, a separate study conducted by Stein et al. (2022), focusing on the effect of pressure on ABE fermentation using *C. acetobutylicum*, concluded that butanol production could be enhanced through overpressure fermentation compared to lower pressure.

In addition, this study investigated the influence of controlled and uncontrolled pH values on butanol production from Enset fiber in the SSF process. The results showed that controlling the pH above 5.0 was not essential to achieve optimal butanol production in the SSF process from Enset fiber. In this study, butanol concentration decreased by 18% in pH-controlled fermentation compared to pH-uncontrolled fermentation, this could be due to the influence of pH value on the efficiency of the cellulase enzyme. Previous research has shown that the optimal pH range for commercial cellulase is typically between 4.8 and 5.5 (Balsan et al. 2012; Wang et al. 2019). However, in this study, during the SSF process in the pH-controlled fermentation, there were fluctuations in the pH values after 8 h, which ranged between 5.5 and 5.96. The primary cause of these fluctuations was the substantial addition of NaOH. When the base pump started adding NaOH to the medium, challenges arose due to the high solid content of the medium and inadequate mixing, impacting the accurate operation of the bioreactor's pH sensor. As a result, the base pump introduced an excessive amount of NaOH, resulting in an elevated pH level. The observed deviation from the optimal pH range reduced enzyme activity, ultimately limiting the release of glucose during pH-controlled fermentation compared to pH-uncontrolled fermentation. In contrast, the pH values in the pH-uncontrolled fermentation after 8 h were between 4.8 and 5.29, which was in the optimal pH range for cellulase enzyme activity. This pH range resulted from the two-phase fermentation of the *Clostridia* strain. Initially, the strain produced acids such as acetic acid and butyric acid, leading to a pH drop to 4.8 during the first phase. Subsequently, the strain transitioned to the solventogenic phase, producing butanol, acetone, and a small amount of ethanol, causing the pH to rose to 5.29 (Lee et al. 2008). Numerous studies have used pH control methods for ABE fermentation to prevent the occurrence of an acid crash, which is often triggered by the presence of inhibitory compounds in the fermentation medium resulting from pretreatment of the lignocellulosic biomass (Yang et al. 2013); however, in this study, there were no inhibitory compounds that could have led to an acid crash. The pH is crucial for SSF process, keeping the pH low after the start of the solventogenic phase might increase butanol production rate and yield but also comes with the costs for pH control.

In this study, the major challenges observed in the bioreactor SSF process were inadequate mixing and reduced butanol productivity. To address these issues, an experiment was conducted using the PSSF method with a 7% (w/v) substrate loading of Enset fiber. The PSSF method alleviated the mass transfer problem by prehydrolysing for 2–3 h before inoculation. This was because the SSF process had poor mixing from the start of fermentation until the Enset fiber was saccharified by the enzyme (Cebreiros et al. 2019). Furthermore, the butanol concentration and productivity were increased by 30% and 27%, respectively, compared to the SSF process with pH control method. This might be due to the initial glucose concentration at the end of the prehydrolysis step, which could facilitate the initial growth of the *C. saccharoperbutylacetonicum* strain (Dong et al. 2016), and the high substrate loading might have contributed to maximizing the butanol concentration. Studies showed that high substrate loading is crucial for large-scale operations to minimize overall production costs and maximize productivity (Weiss et al. 2019); by increasing the substrate loading from 5% (w/v) to 8% (w/v) of biomass, the production cost can be reduced by 19%, as this can lower the volume of downstream processing equipment and the energy consumption that is related to the dilution level (Wingren et al. 2003).

Compared to previous PSSF studies with different biomass and *Clostridial* strains, the highest butanol concentration and yield were observed in Enset fiber than in sugarcane straw (Pratto et al. 2020), corn stover (Wu et al. 2021) and napier grass (He et al. 2017). However, the butanol productivity of this study was lower than that of Dong et al. (2016), who performed the PSSF process using *C. saccharobutylicum* from corn stover at an SSF temperature of 37 °C. This could be due to the lower working temperature of the SSF process in this study, which slowed down enzyme activity, or the inhibitory concentration of butanol on the strain (Soni et al. 1987). To enhance the butanol productivity of this study, utilizing a strain with high-temperature and solvent tolerance (Zhao et al. 2019) or cold-active cellulase () may enable a more effective PSSF process from Enset fiber.Hydrogen production from Enset fiber using SSF process.

Producing hydrogen and butanol from affordable materials such as Enset fiber is essential to ensure the sustainability of the biofuel industry. The choice between butanol or hydrogen as a biofuel depends on application, cost considerations, and environmental sustainability (Ahmed et al. 2021; Dahman et al. 2019). In this study, butanol and hydrogen were simultaneously produced by the SSF process from Enset fiber using *C. saccharoperbutylacetonicum*. However, the fermentation conditions must be adjusted separately to achieve maximum yields for both products. This is because fermentation conditions affect cell metabolism and can potentially shift the metabolic pathway in favor of hydrogen or butanol production (Nakayama et al. 2013). In addition, it has been shown that the amount of hydrogen and butanol produced in the *Clostridia* strain is influenced by factors such as electron flow and the balance of reducing equivalents in the metabolic pathway, leading to a competitive relationship between the two products (Wu et al. 2017).

Temperature is a key factor in the SSF process, as it influences both the growth of the cells and the enzyme activity in their metabolism. The results indicated that higher temperatures (35 and 37 °C) improved hydrogen production compared to lower temperature (30 °C); however, butanol production decreased with increasing temperature in the SSF process using *C. saccharoperbutylacetonicum*. A possible explanation could be that the cells grew faster at the higher temperature in the initial growth phase (Yadav et al. 2021), which coincided with the highest hydrogen production rate in the acidogenic phase observed in this study. The result of this study was similar to those of Alalayah et al. (2008) and Yadav et al. (2021), who investigated the influence of temperature on hydrogen production in ABE fermentation using *C. saccharoperbutylacetonicum* and found the highest hydrogen values at 37 °C in both studies.

Another factor that affected hydrogen production in the SSF process was the initial pH value of the medium, which was influenced by the buffers in the medium and the metabolites in the cell growth phase. As *C. saccharoperbutylacetonicum* initiated growth, it began by producing acetic acid, butyric acid, and hydrogen. The buildup of organic acids resulted in a decrease in the pH of the medium and created stressful conditions for cell growth. Subsequently, the culture converted these acids into solvents such as butanol, acetone, and ethanol as it entered the stationary phase, increasing in pH value. Under optimal conditions favorable for cell growth, these phase changes represent the natural pathway for ABE fermentation (Lee et al. 2008). However, in the presence of inhibitors or unfavorable conditions, the cells struggle to survive, leading to increased acid production and eventual cell death (Li et al. 2020). In this study, the pH profile of the SSF process at initial pH of 6.0, 7.0, and 8.0 followed a similar phase change and showed no significant difference in hydrogen and butanol production. However, at initial pH of 5.0 and 9.0, the strain could not enter the solventogenic phase, indicating that lower and higher initial pH values inhibited the cell. A study using the same medium and strain for ABE fermentation as in this study found that hydrogen production was maximized in the initial pH range of 7.0–8.5 (Ferchichi et al. 2005). In contrast, other studies suggested an optimal initial pH of 6.5, and beyond this point, hydrogen production decreased, although they used a different medium (Singh et al. 2019; Alalayah et al. 2008).

In addition, this study examined the effect of hydrogen partial pressure on hydrogen production and found that lower hydrogen partial pressure was beneficial for maximizing hydrogen production. The results of bottle experiments showed that hydrogen production was increased by 21.5% at lower hydrogen partial pressure compared to higher values. To validate the bottle experiment results and maximize hydrogen production, a scale up SSF process without a pH control system was implemented at the optimal process parameters with a temperature of 37 °C, an initial pH of 8.0 and under atmospheric pressure. The results confirmed that hydrogen production increased by 79.7% compared to the SSF process (pH-uncontrolled) at 30 °C, with an initial pH of 6.8 and under overpressure. However, butanol production was reduced by 21.4% at optimum process conditions for hydrogen production. This study achieved a higher hydrogen yield of 198.27 mL/g-Enset fiber compared to the previous study by Li et al. (2007), which reported a hydrogen yield of 68 mL/g-biomass from steam-exploded straw using the SSF process with the *C. butyricum* AS1.209 strain. Several studies have shown that hydrogen partial pressure is an essential factor in maximizing hydrogen production; it could also potentially inhibit cell growth and limit the thermodynamics of the process, depending on the microorganisms involved (Yerushalmi et al. 1985; Stein et al. 2022). Electrons play a crucial role in the *Clostridial* metabolic pathways, and the strain can release excess electrons as hydrogen gas to balance the nicotinamide adenine dinucleotide (NADH/NAD^+^) ratio. However, when the hydrogen gas levels are high, it becomes more difficult for the bacteria to continue producing more hydrogen gas; this means the bacteria have too many electrons and insufficient energy, which can stop their growth and activity. As a consequence of this electron backlog, the strain utilises alternative pathways in which electrons are transferred from NADH to ferredoxin, which can serve as an electron carrier to reduce NADP^+^ to NADPH, which facilitates butanol production (Wu et al. 2017; Vamsi Krishna et al. 2022; Foulquier et al. 2022). For this reason, removing hydrogen gas from the medium might help the strain to produce more hydrogen.

## Conclusions

This study demonstrated the potential of Enset fiber as a feedstock for hydrogen and butanol production in the SSF process. Through optimization of SSF process parameters using *C. saccharoperbutylacetonicum* strain, we achieved high yields and concentrations of both biofuels under different conditions. Furthermore, a scalable process for both products was developed to achieve similar results to bottle-scale experiments, indicating potential applicability on an industrial scale. This is the first study to use this strain and substrate combination for the SSF process, and it contributes to the development of sustainable energy sources. However, -further research on the 5L, 10L bioreactors and pilot scale processes is necessary. Additionally, a comprehensive feasibility study and environmental impact assessment, are required to make industrial-scale butanol and hydrogen production feasible using the SSF process from Enset fiber.Materials and methods.

### Enset fiber preparation and dilute alkali pretreatment method

Enset fiber was obtained from a privately owned Enset plantation in Wolkite, Ethiopia. The Enset fiber was prepared and pretreated according to the methods previously described by Seid et al. (2022). First, the sample was manually cut into 6 cm pieces using scissors, then sun-dried for 4 days. The dried sample was pulverized using a knife mill and sieved to 2 mm particle size. The resulting dry powder was subjected to a dilute alkali pretreatment process. In this process, the sample was placed in an Erlenmeyer flask and mixed with 2% (w/v) NaOH, then autoclaved at 121 °C for 20 min. After autoclaving, the sample was cooled, centrifuged at 4700 × g for 30 min, and filtered. The residue was repeatedly washed with deionized water, the pH was adjusted, and filtered again using a muslin cloth. According to the NREL standard (NREL/TP-510-42621), the residue sample was dried at 105 °C for 24 h in a convection oven to determine its moisture content (Sluiter et al. 2008) and then subjected to the SSF process.

### Bacterial strain and culture medium

*Clostridium saccharoperbutylacetonicum* DSM 14923 was obtained from the German Collection of Microorganisms and Cell Cultures (DSMZ, Braunschweig, Germany). To prepare the inoculum, glycerol stock of the strain (1 mL) was added to 50 mL of anaerobic TGY medium, and the culture was incubated at 30 °C until the optical density (OD_600_) reached 1.0–1.2. TGY medium contained 30 g/L tryptone, 20 g/L glucose, 10 g/L yeast extract, and 0.4 g/L cysteine-HCl·H_2_O. The main fermentation medium comprises 1% (v/v) P2 stock solutions, which was prepared separately, buffer stock solution (50 g/L KH_2_PO_4_, 50 g/L K_2_HPO_4_, and 220 g/L CH_3_COONH_4_), mineral stock solution (20 g/L MgSO_4_·7H_2_O, 1 g/L MnSO_4_·H_2_O, 1 g/L FeSO_4_·7H_2_O, and 1 g/L NaCl), and vitamin stock solution (0.1 g/L para-aminobenzoic acid, 0.1 g/L thiamin, and 0.001 g/L biotin) (Yao et al. 2017). All stock solutions were filter-sterilized and anaerobized using N_2_ gas.

### Preparation of SSF medium from pretreated Enset fiber for the bottle experiment

To prepare the SSF medium, different amounts of pretreated Enset fiber were added as the sole carbon source into 250 mL Duran pressure plus bottles, each containing a total volume of 50 mL of medium. Subsequently, 1 g/L of yeast extract, 1 mL of 1 g/L resazurin and 0.4 g/L of cysteine-HCl·H_2_O were added, and the initial pH was adjusted using NaOH/H_3_PO_4_ to different pH values. The bottles were sealed using a rubber stopper and a cap, and anaerobized. The anaerobization process was conducted by flushing the bottles with N₂ gas using needles connected to the gas lines. Then, the bottles were autoclaved at 121 °C for 20 min. Following autoclaving, 1% (v/v) of each P2 stock solutions and 5 mL cellulase-water mixed solution (Cellic CTec2) (Sigma-Aldrich Chemie GmbH, Hamburg, Germany) at varying cellulase loading were added. The bottles were inoculated with 5% (v/v) active *C. saccharoperbutylacetonicum* culture and incubated in Infors Thermotron incubators (Infors AG, Bottmingen, Switzerland) for 72 h at different temperatures and agitation speeds. In each experiment, the pressure inside the bottles was measured with a GMH 3100 Series manometer (Greisinger, Mainz, Germany), and samples of 3 mL of gas and 1.5 mL of liquid were collected using needles and syringes. In addition, the saccharification experiment was conducted as a control which was without adding inoculum, and the medium was prepared following the procedure outlined by Seid et al. (Seid et al. 2022).Influence of SSF process parameters on butanol and hydrogen production in the bottle experiment.

Preliminary experiments were carried out to find out which temperature ranges were suitable for the cultivation of *C. saccharoperbutylacetonicum* in the SSF process using Enset fiber at various temperatures, including 28, 30, 33, 35, 37, 40, 45, and 50 °C. It was performed with a substrate loading of 5% (w/v), which was 2.5 g pretreated Enset fiber (50 g/L), a cellulase loading of 24 FPU/g, an initial pH of 6.8 and an agitation speed of 130 rpm.

The optimization experiment was conducted using the One-Factor-at-a-Time (OFAT) approach, where each parameter was varied individually while keeping others constant. First, the influences of agitation speed, cellulase loading and substrate loading on butanol production in the SSF process were investigated at a constant temperature of 30 °C and an initial pH of 6.8 (Yao et al. 2017). Initially, the effects of agitation were tested at different speeds (0, 100, 130 rpm) while maintaining a constant substrate loading of 5% (w/v) and a cellulase loading of 24 FPU/g. After determining an optimal agitation speed (100 rpm), experiments were conducted to examine the effect of different cellulase loadings (9, 16, 24, 30 FPU/g) while maintaining a constant substrate loading of 5% (w/v). Subsequently, the effects of substrate loading on butanol production were tested at different substrate loadings of Enset fiber (2, 3, 5, 7% (w/v)) while maintaining constant agitation (100 rpm) and cellulase loading (16 FPU/g). Finally, a validation experiment was performed under optimal conditions with an agitation speed of 100 rpm, a cellulase loading of 16 FPU/g and a substrate loading of 5% (w/v). As a control experiment, optimization of the saccharification process was performed in a similar manner to determine how changes in process parameters affect the efficiency of enzymatic hydrolysis.

In addition, the influence of temperature, initial pH, and hydrogen partial pressure on hydrogen production in the SSF process were examined under optimal process parameters, which included an agitation speed of 100 rpm, a cellulase loading of 16 FPU/g, and a substrate loading of 5% (w/v) Enset fiber. First, to study the effects of temperature on hydrogen production, bottles were placed in incubators set at various temperatures (30, 35, 37 °C) while maintaining a constant initial pH of 6.8 in closed bottles. Following the selection of the best temperature (37 °C) for hydrogen production, the effects of initial pH were examined by adjusting the SSF medium to the initial pH (5.0, 6.0, 7.0, 8.0, and 9.0) while maintaining a constant temperature. Afterwards, the effects of hydrogen partial pressure were determined at a constant temperature of 37 °C and an initial pH of 8.0. The following experimental setup was established to investigate the effect of hydrogen partial pressure on hydrogen production, the 250 mL bottles containing the fermentation medium were connected to empty bottles (anaerobized 1-L and 2-L bottles) by tube. This setup allowed the hydrogen-rich gas produced by fermentation to flow from the smaller bottles to the larger ones. All experiments were performed in triplicate and lasted 72 h.

### Bioreactor setup for the SSF process from pretreated Enset fiber

A 2.5 L bioreactor (Minifors, Infors HT, Bottmingen, Switzerland) with two six-bladed Rushton turbine impellers was used for the SSF process. The impellers had a diameter of 4.6 cm and a width of 1.1 cm and positioned 6.0 cm apart on the stirrer. For the SSF medium preparation in the bioreactor, 50 g of pretreated Enset fiber with a substrate loading of 5% (w/v) was added to the bioreactor as the sole carbon source, and the medium had a total volume of 1 L. The bioreactor was filled with liquids containing 1 g/L yeast extract and 1 mL of 1 g/L resazurin and autoclaved. After autoclaving, all tubes and the bioreactor were sealed airtight and anaerobized overnight using filter-sterilized N_2_ gas while being mixed at a stirring speed of 500 rpm. After anaerobization, the bioreactor was supplemented with 1% (v/v) of each P2 stock solution and 0.4 g/L of cysteine-HCl·H_2_O using needles and syringes. For the main fermentation, the temperature was adjusted to the desired level, and the stirring speed was reduced to 150 rpm (based on preliminary experiments in the bioreactor). Subsequently, the N_2_ gas flow was stopped, 10 mL of a cellulase-water mixed solution (Cellic CTec2) with a cellulase loading of 16 FPU/g was added to the bioreactor and inoculated with 5% (v/v) active *C. saccharoperbutylacetonicum* culture. 5 mL gas and 2 mL liquid samples were collected and analyzed.

The bioreactor setup varied depending on the type of fermentation. For butanol production, fermentations were performed at a temperature of 30 °C, with an initial pH of 6.8 and under overpressure, with the gases partially kept in the bioreactor. The bioreactor outlet tube was connected to the pressure relief valve (V07, IMI Norgren, Lichfield, United Kingdom) and set to an absolute pressure of 1.55 bar. After the pressure reached 1.55 bar, the valve was opened, and the gases produced were passed through BlueVCount (BlueSens gas sensor GmbH, Herten, Germany) to measure the volume of gases accumulated in the gas bags. In addition, for the pH-controlled experiment, the pH of the medium was controlled at pH ≥ 5.0 by using the bioreactor's base pump containing 4 M NaOH (Feng et al. 2020). For hydrogen production, fermentation was performed at 37 °C, with an initial pH of 8.0 and under atmospheric pressure, releasing the gas from the bioreactor. In this fermentation, the bioreactor outlet tube was directly connected to the BlueVCount and accumulated in the gas bags. All experiments were incubated for 120 h and performed in duplicate.

### PSSF process from pretreated Enset fiber in the bioreactor

The PSSF medium was prepared similarly to the SSF medium except for the substrate loading, which was 7% (w/v) of pretreated Enset fiber (70 g/L), and operating conditions. Before inoculation with an active preculture of *C. saccharoperbutylacetonicum*, the media was hydrolyzed for 2 h. The prehydrolysis experiment was started by adjusting the pH to 5.0 and the temperature to 40 °C. Then, the cellulase enzyme was added at 16 FPU/g cellulase loading and stirred at 500 rpm. Afterwards, the temperature and stirrer speed were minimized to 30 °C and 150 rpm, respectively, and the initial pH was adjusted to 6.8, then inoculated with 5% (v/v) active preculture of *C. saccharoperbutylacetonicum*. During the fermentation, the pH was controlled with 4M NaOH to pH ≥ 5.0 and the pressure to 1.5 bar. The experiment was incubated for 120 h and performed in duplicate.

### Analytical methods

The pH of the liquid sample was analysed by using Profilab pH 597 (Xylem Analytics, Weilheim, Germany) pH meter for bottle experiment. Monomeric sugars and fermentation metabolites in all samples were analysed by high-performance liquid chromatography (HPLC) in an 1100 Series System (Agilent Technologies, Waldbronn, Germany), following the method described by Seid et al. (2022). The gas pressure developed during fermentation was measured using a manometer, and the gas composition was determined using the Micro GC Fusion^®^ gas analyzer (Inficon, Bad Ragaz, Switzerland) equipped with PLOT and WCOT columns and utilizing Argon and Helium as carrier gases (Seid et al. 2023). The volume of gas generated from the bioreactor was measured using BlueVCount. The moles of gas for each experiment were calculated using the ideal gas law. The total moles of the gas in the bioreactor were determined by summing the moles of gas inside the bioreactor and the moles of gas passing through the BlueVCount.

## Supplementary Information

Additional file 1: Table S1. Output from ANOVA analysis in OriginPro 2021; Effect of agitation speed on butanol production in the SSF process from Enset fiber. Table S2. Effect of temperature on hydrogen and biobutanol production in the SSF process from Enset fiber in bottles. Table S3. Effect of initial pH on hydrogen and biobutanol production in the SSF process from Enset fiber in bottles. Table S4. Output from ANOVA analysis in OriginPro 2021; Effect of gas release strategy on hydrogen production in the SSF process from Enset fiber. Table S5. Effect of hydrogen partial pressure on hydrogen and biobutanol production in the SSF process from Enset fiber in bottles. Table S6. Carbon and electron balance calculation for the SSF process from Enset fiber at atmospheric pressure, initial pH of 8.0 (uncontrolled) and 37 °C. Table S7. Carbon and electron balance calculation and conversion factor for the SSF process from Enset fiber at 0.55 bar overpressure, initial pH of 6.9 (uncontrolled) and 30 °C.

## Data Availability

Not applicable.

## Abbreviations

ABE: Acetone-butanol-ethanol
IEA: International energy agency
PSSF: Prehydrolysis simultaneous saccharification and fermentation
SHF: Separate hydrolysis and fermentation
SSF: Simultaneous saccharification and fermentation
