# Supplementary material for: Stirring the hydrogen and butanol production from Enset fiber via simultaneous saccharification and fermentation (SSF) process
Source: Bioresour Bioprocess. 2024 Oct 10;11(1):96. doi: 10.1186/s40643-024-00809-w (PMC11466926; doi:10.1186/s40643-024-00809-w)
Supplement: Supplementary file 1 — Additional file 1: Table S1. Output from ANOVA analysis in OriginPro 2021; Effect of agitation speed on butanol production in the SSF process from Enset fiber. Table S2. Effect of temperature on hydrogen and biobutanol production in the SSF process from Enset fiber in bottles. Table S3. Effect of initial pH on hydrogen and biobutanol production in the SSF process from Enset fiber in bottles. Table S4. Output from ANOVA analysis in OriginPro 2021; Effect of gas release strategy on hydrogen production in the SSF process from Enset fiber. Table S5. Effect of hydrogen partial pressure on hydrogen and biobutanol production in the SSF process from Enset fiber in bottles. Table S6. Carbon and electron balance calculation for the SSF process from Enset fiber at atmospheric pressure, initial pH of 8.0 (uncontrolled) and 37 °C. Table S7. Carbon and electron balance calculation and conversion factor for the SSF process from Enset fiber at 0.55 bar overpressure, initial pH of 6.9 (uncontrolled) and 30 °C. [file 40643_2024_809_MOESM1_ESM.docx]

**Stirring** **the Hydrogen and Butanol production from Enset Fiber via Simultaneous Saccharification and Fermentation (SSF) process**

Nebyat Seid^1, 2, *^, Lea Wießner^1^, Habibu Aliyu^3^ and Anke Neumann^1, *^

^1^Electrobiotechnology, Institute of Process Engineering in Life Science 2, Karlsruhe Institute of Technology (KIT), 76131 Karlsruhe, Germany

^2^School of Chemical and Bio Engineering, Addis Ababa Institute of Technology, Addis Ababa University, P.O.B: 1176, Addis Ababa, Ethiopia

^3^Institute for Biological Interfaces 5, Karlsruhe Institute of Technology (KIT), 76344 Karlsruhe, Germany

^*^Correspondence: [anke.neumann@kit.edu](mailto:anke.neumann@kit.edu) or nebyatabdu@gmail.com

**Table S1** Output from ANOVA analysis in OriginPro 2021; Effect of agitation speed on butanol production in the SSF process from Enset fiber


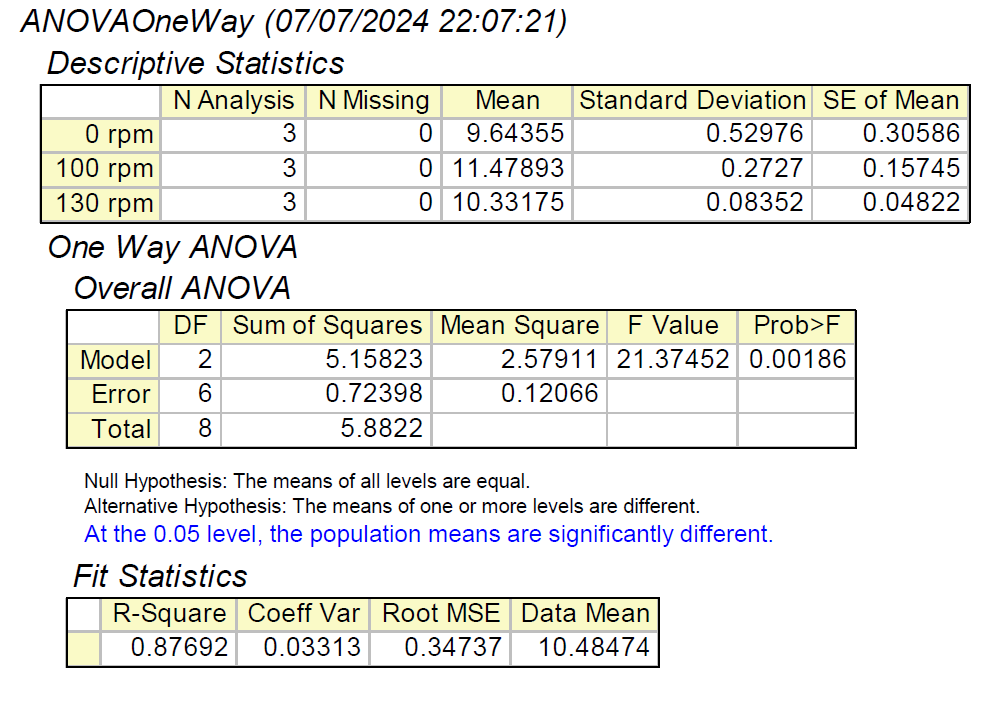


**Table S2** Effect of temperature on hydrogen and biobutanol production in the SSF process from Enset fiber in bottles ^a, b,c^

| Temp. (°C) | Final glucose concentration (g/L) | Butanol concentration (g/L) | Butanol yield (g/g) | Butanol productivity (g/(L h)) | Hydrogen (mmol) | Hydrogen yield (mL/g) | Maximum hydrogen production rate (mmol/h) |
| --- | --- | --- | --- | --- | --- | --- | --- |
| 30 | 2.69 ± 0.98 | 11.25 ± 0.05 | 0.23 | 0.17 | 12.03 ± 0.23 | 107.79 | 0.35 |
| 35 | 0.50 ± 0.04 | 10.59 ± 0.10 | 0.21 | 0.15 | 14.67 ± 0.13 | 131.44 | 0.61 |
| 37 | 5.86 ± 0.81 | 8.30 ± 0.39 | 0.17 | 0.12 | 14.90 ± 0.13 | 133.50 | 0.45 |

^a^ All calculations accounted for 2.5 g Enset fiber in 50 ml medium at 72 hours fermentation period; ^b^ Conversion of mmol to ml of hydrogen was carried out in accordance with the ideal gas law under standard temperature and pressure conditions.; ^c^ Values are means from triplicate bottles.

**Table S3** Effect of initial pH on hydrogen and biobutanol production in the SSF process from Enset fiber in bottles ^a, b,c^

| Initial pH | Final glucose concentration (g/L) | Butanol concentration (g/L) | Butanol yield (g/g) | Butanol productivity (g/(L h)) | Hydrogen (mmol) | Hydrogen yield (mL/g) | Maximum hydrogen production rate (mmol/h) |
| --- | --- | --- | --- | --- | --- | --- | --- |
| 5.0 | 38.00 ± 0.45 | 0.24 ± 0.20 | 0.005 | 0.003 | 1.74 ± 0.16 | 15.59 | 0.10 |
| 6.0 | 5.36 ± 0.62 | 8.70 ± 0.13 | 0.174 | 0.121 | 15.48 ± 0.34 | 138.70 | 0.39 |
| 7.0 | 5.76 ± 0.03 | 8.74 ± 0.26 | 0.175 | 0.121 | 15.54 ± 0.30 | 139.24 | 0.50 |
| 8.0 | 3.5 ± 0.66 | 9.36 ± 0.20 | 0.187 | 0.130 | 15.52 ± 0.07 | 139.06 | 0.53 |
| 9.0 | 30.39 ± 1.46 | 1.81 ± 0.70 | 0.036 | 0.025 | 5.33 ± 0.87 | 47.76 | 0.32 |

^a^ All calculations accounted for 2.5 g Enset fiber in 50 ml medium at 72 hours fermentation period; ^b^ Conversion of mmol to ml of hydrogen was carried out in accordance with the ideal gas law under standard temperature and pressure conditions.; ^c^ Values are means from triplicate bottles.

**Table S4** Output from ANOVA analysis in OriginPro 2021; Effect of gas release strategy on hydrogen production in the SSF process from Enset fiber


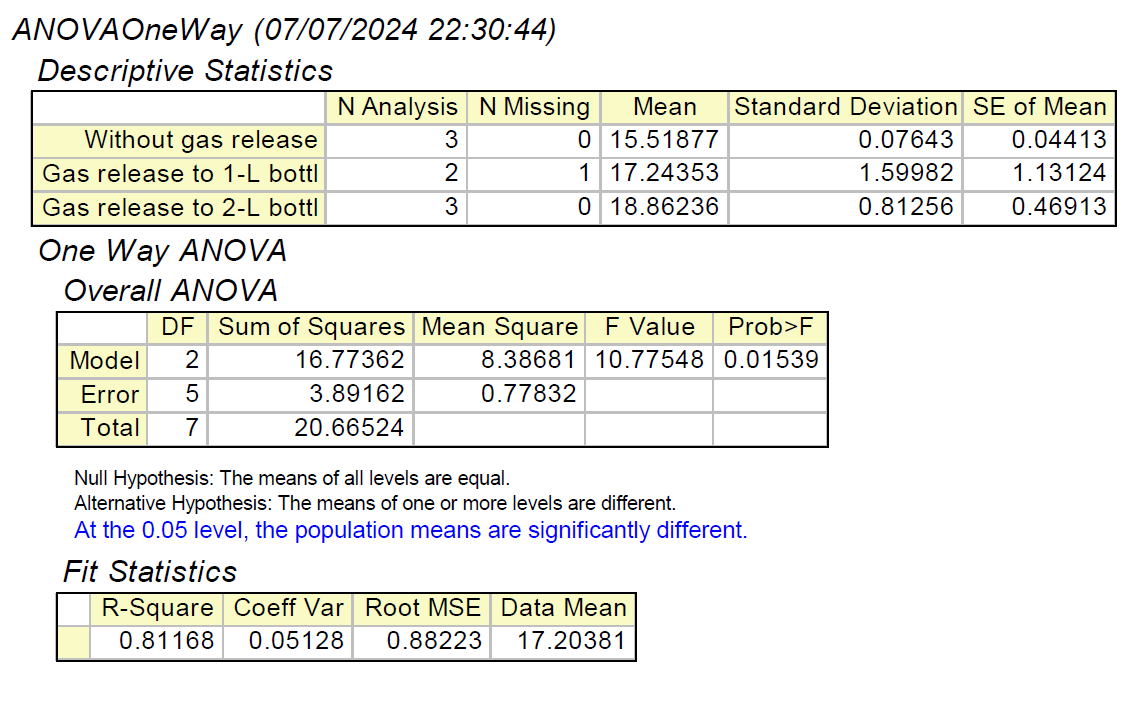


**Table S5** Effect of hydrogen partial pressure on hydrogen and biobutanol production in the SSF process from Enset fiber in bottles ^a, b,c^

| Gas release strategy | Final glucose concentration (g/L) | Butanol concentration (g/L) | Hydrogen (mmol) | Hydrogen yield (mL/g) | Maximum hydrogen production rate (mmol/h) |
| --- | --- | --- | --- | --- | --- |
| Without gas release | 3.50 ± 0.66 | 9.36 ± 0.20 | 15.52 ± 0.07 | 139.06 | 0.53 |
| Gas release to 1-L bottle | 2.40 ± 0.45 | 9.23 ± 0.93 | 17.24 ± 1.59 | 154.47 | 0.41 |
| Gas release to 2-L bottle | 0.19 ± 0.08 | 9.65 ± 0.17 | 18.86 ± 0.81 | 168.99 | 0.59 |

^a^ All calculations accounted for 2.5 g Enset fiber in 50 ml medium at 72 hours fermentation period; ^b^ Conversion of mmol to ml of hydrogen was carried out in accordance with the ideal gas law under standard temperature and pressure conditions.; ^c^ Values are means from triplicate bottles.**Table S6** Carbon and electron balance calculation for the SSF process from Enset fiber at atmospheric pressure, initial pH of 8.0 (uncontrolled) and 37 °C.

| Compound | Chemical formula | Molecular weight (g/mol) | Concent-ration (g/L) | Mole of compound (mol) | mol Carbon /mol | Carbon per compound (mol) | e-mol /mol | e-mol  per compound (mol) | e-mol yield | e-mol yield (%) |
| --- | --- | --- | --- | --- | --- | --- | --- | --- | --- | --- |
| ***Initial Substrates*** | | | | | | | | | | |
| Cellulose | C_12_H_20_O_10_ | 324.28 | 50 | 0.154 | 12 | 1.850 | 48 | 7.401 | 0.979 | 97.92 |
| Acetate | C_2_H_4_O_2_ | 60 | 1.179 | 0.019 | 2 | 0.039 | 8 | 0.157 | 0.021 | 2.08 |
| **Sum of substrates** | | | | | | 1.889 |  | 7.558 |  |  |
| ***Final products*** | | | | | | | | | | |
| Hydrogen | H_2_ |  |  | 0.443 |  |  | 2 | 0.886 | 0.117 | 11.72 |
| CO2 | CO_2_ |  |  | 0.524 | 1 | 0.524 | 0 | 0 | 0 | 0 |
| Butanol | C_4_H_10_O | 74 | 8.922 | 0.121 | 4 | 0.482 | 24 | 2.894 | 0.383 | 38.29 |
| Acetone | C_3_H_6_O | 58 | 6.038 | 0.104 | 3 | 0.312 | 16 | 1.666 | 0.220 | 22.04 |
| Butyrate | C_4_H_8_O_2_ | 88 | 0.619 | 0.007 | 4 | 0.028 | 20 | 0.141 | 0.019 | 1.86 |
| Ethanol | C_2_H_6_O | 46 | 0.288 | 0.006 | 2 | 0.013 | 12 | 0.075 | 0.010 | 0.99 |
| Acetate | C_2_H_4_O_2_ | 60 | 1.898 | 0.032 | 2 | 0.063 | 8 | 0.253 | 0.033 | 3.35 |
| Glucose | C_6_H_12_O_6_ | 180 | 0.797 | 0.004 | 6 | 0.027 | 24 | 0.106 | 0.014 | 1.41 |
| Other sugars (xylose, mannose, and galactose) | C_5_H_10_O_5_ | 150 | 0.908 | 0.006 | 5 | 0.030 | 20 | 0.121 | 0.016 | 1.60 |
| **Sum of products** | | | | | | 1.479 |  | 6.141 |  |  |
| **Carbon recovery (%)*** | | | | | | **78.29** |  |  |  |  |
| **e-mol recovery (%)*** | |  |  |  |  |  |  | **81.26** |  |  |

*Carbon & e-mol recovery (%) = (Sum of products/ Sum of substrate) × 100%

**Table S7** Carbon and electron balance calculation and conversion factor for the SSF process from Enset fiber at 0.55 bar overpressure, initial pH of 6.9 (uncontrolled) and 30 °C.

| Compound | Chemical formula | Molecular weight (g/mol) | Concent-ration (g/L) | Mole of compound (mol) | mol Carbon /mol | Carbon per compound (mol) | e-mol /mol | e-mol  per compound (mol) | e-mol yield | e-mol yield (%) |
| --- | --- | --- | --- | --- | --- | --- | --- | --- | --- | --- |
| ***Initial Substrates*** | | | | | | | | | | |
| Cellulose | C_12_H_20_O_10_ | 324.28 | 50 | 0.154 | 12 | 1.850 | 48 | 7.401 | 0.969 | 96.91 |
| Acetate | C_2_H_4_O_2_ | 60 | 1.77 | 0.029 | 2 | 0.059 | 8 | 0.236 | 0.031 | 3.09 |
| **Sum of substrates** | | | | | | 1.909 |  | 7.637 |  |  |
| ***Final products*** | | | | | | | | | | |
| Hydrogen | H_2_ |  |  | 0.246 |  |  | 2 | 0.492 | 0.064 | 6.44 |
| CO2 | CO_2_ |  |  | 0.467 | 1 | 0.467 | 0 | 0 | 0 | 0 |
| Butanol | C_4_H_10_O | 74 | 11.352 | 0.153 | 4 | 0.614 | 24 | 3.682 | 0.482 | 48.21 |
| Acetone | C_3_H_6_O | 58 | 3 | 0.052 | 3 | 0.155 | 16 | 0.828 | 0.108 | 10.84 |
| Butyrate | C_4_H_8_O_2_ | 88 | 1.336 | 0.015 | 4 | 0.061 | 20 | 0.304 | 0.039 | 3.98 |
| Ethanol | C_2_H_6_O | 46 | 0.332 | 0.007 | 2 | 0.014 | 12 | 0.087 | 0.011 | 1.13 |
| Acetate | C_2_H_4_O_2_ | 60 | 0.612 | 0.010 | 2 | 0.020 | 8 | 0.082 | 0.011 | 1.07 |
| Glucose | C_6_H_12_O_6_ | 180 | 1.779 | 0.009 | 6 | 0.059 | 24 | 0.237 | 0.031 | 3.11 |
| Other sugars (xylose, mannose, & galactose) | C_5_H_10_O_5_ | 150 | 1.001 | 0.007 | 5 | 0.033 | 20 | 0.133 | 0.017 | 1.75 |
| **Sum of products** | | | | | | 1.424 |  | 5.844 |  |  |
| **Carbon recovery (%)*** | | | | | | **74.59** |  |  |  |  |
| **e-mol recovery (%)*** | | | | | | | | **76.52** |  |  |

*Carbon & e-mol recovery (%) = (Sum of products/ Sum of substrate) × 100%
